# Annotation and analysis of a large cuticular protein family with the R&R Consensus in *Anopheles gambiae*

**DOI:** 10.1186/1471-2164-9-22

**Published:** 2008-01-18

**Authors:** R Scott Cornman, Toru Togawa, W Augustine Dunn, Ningjia He, Aaron C Emmons, Judith H Willis

**Affiliations:** 1 Department of Cellular Biology, University of Georgia, Athens, GA 30602 USA; 2 The Key Laboratory of Sericulture, Southwest University, Chongqing 400715, PR China

## Abstract

**Background:**

The most abundant family of insect cuticular proteins, the CPR family, is recognized by the R&R Consensus, a domain of about 64 amino acids that binds to chitin and is present throughout arthropods. Several species have now been shown to have more than 100 CPR genes, inviting speculation as to the functional importance of this large number and diversity.

**Results:**

We have identified 156 genes in *Anopheles gambiae *that code for putative cuticular proteins in this CPR family, over 1% of the total number of predicted genes in this species. Annotation was verified using several criteria including identification of TATA boxes, INRs, and DPEs plus support from proteomic and gene expression analyses. Two previously recognized CPR classes, RR-1 and RR-2, form separate, well-supported clades with the exception of a small set of genes with long branches whose relationships are poorly resolved. Several of these outliers have clear orthologs in other species. Although both clades are under purifying selection, the RR-1 variant of the R&R Consensus is evolving at twice the rate of the RR-2 variant and is structurally more labile. In contrast, the regions flanking the R&R Consensus have diversified in amino-acid composition to a much greater extent in RR-2 genes compared with RR-1 genes. Many genes are found in compact tandem arrays that may include similar or dissimilar genes but always include just one of the two classes. Tandem arrays of RR-2 genes frequently contain subsets of genes coding for highly similar proteins (sequence clusters). Properties of the proteins indicated that each cluster may serve a distinct function in the cuticle.

**Conclusion:**

The complete annotation of this large gene family provides insight on the mechanisms of gene family evolution and clues about the need for so many CPR genes. These data also should assist annotation of other *Anopheles *genes.

## Background

**Figure 1:**
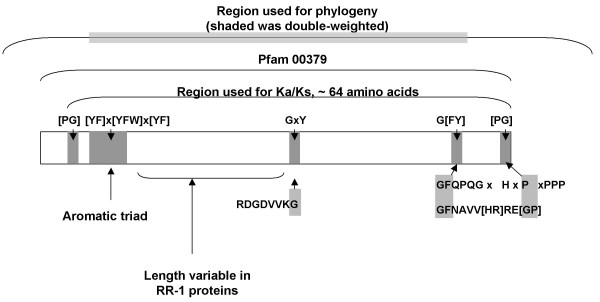
**Features of the R&R Consensus in *An. gambiae *and their relationship to the pfam00379 motif**. The longest region that generally could be aligned across *An. gambiae *CPRs is shown beginning with a proline or glycine two or three sites prior to the aromatic triad and ending with a proline or glycine eight positions C-terminal to the final invariant glycine. Our alignment of this region was 65 positions long for aligned RR-2 genes, but the complete alignment was 83 positions long due to length variation in RR-1 genes. The pfam00379 alignment includes seven additional positions N-terminal to our alignment. The region used in the phylogeny extended further in the N-terminal and C-terminal directions as described in the text. The shaded region on the top line was double-weighted because this encompasses the principal features of the R&R Consensus that are present in virtually all *An. gambiae *CPRs.

Arthropod cuticle consists predominantly of chitin fibers embedded in a protein matrix [1]. While chitin is a simple polymer of N-acetylglucosamine, there is a large number of cuticular proteins (see [2,3] for review). The vast majority of cuticular protein sequences presently available belong to a family with the R&R Consensus, first identified by Rebers and Riddiford [4]. An extended version of the original Consensus has been shown to bind to chitin [5,6], and the conformation it may adopt has been modeled [7,8]. Throughout this paper, we will use the term, R&R Consensus, to refer to the extended Consensus and CPR to refer to the family of genes/proteins with this Consensus. The Consensus, with about 64 amino acids, almost always begins near a triad of aromatic residues (Y/F-x-Y/F/W-x-Y/F) and terminates shortly after a uniformly conserved G-F/Y (Figure 1).

While the R&R Consensus is conserved across arthropods, its location within a cuticular protein and the nature of the regions that flank it are highly variable. Understanding of the role of these proteins in forming the insect exoskeleton and other cuticular structures will be facilitated by defining all of the cuticular proteins of a single species. Accounts of the cuticular proteins with the R&R Consensus have now been published for 28 proteins from *Apis mellifera *[9] and for 101 from *Drosophila melanogaster *[10]. Also 102 CPR proteins have been identified in the genome of *Tribolium castaneum *(Beeman and Willis, unpublished observations). These annotations depended in large part on computerized genome annotation and were not systematically verified at the mRNA or protein level.

In the present study, we have carried out an exhaustive manual annotation of the CPR family of *An. gambiae *based on the whole genome sequence of the PEST strain [11]. These annotations are being facilitated and verified by a proteomics analysis of cuticles [[12], He unpublished observations] and accompanied by an analysis of gene expression with real-time RT-PCR [13]. In addition, ambiguous gene models have been confirmed or revised by sequencing RT-PCR or RACE products. This work has identified 156 genes coding for CPR proteins. Hence over 1% of the genes of *An. gambiae *are devoted to just this one family of cuticular proteins.

An investigation of cuticular proteins in *An. gambiae *carried out prior to whole genome sequencing was particularly informative for the present annotation study. Dotson et al. [14] sequenced a 17.4 kb insert in a genomic library constructed from the Sua strain. This region had three CPR genes that were at least 98% identical in their coding regions, yet differed in 5' and 3' UTRs as well as their introns. Hence, the lesson learned was that virtually identical genes can reside in compact tandem arrays, yet can be recognized as distinct and not an assembly artefact because of the differences in the non-coding regions associated with them.

CPR proteins can be divided into groups according to which variant of the extended R&R Consensus they possess. Two major groups have been named RR-1 and RR-2 while a third group (RR-3) has been identified but from only a small number of sequences [15,16]. It is unclear whether RR-3s are an evolutionarily distinct group; for the present analysis we include RR-3 genes within the RR-1 class. A Hidden Markov Model can be employed at the cuticle DB web server [17] to assign proteins as RR-1 or RR-2 [10]. Our analysis confirms that the bulk of RR-1 and RR-2 proteins form non-overlapping clades in *An. gambiae*, separated by a small set of long-branch RR-1, RR-2, and RR-3 proteins that are probably an artificial group. In addition to assembling information that supports annotation, we have analyzed the structure of these clades, examined patterns of molecular evolution, compared the amino acid composition of the different proteins and identified characteristics of each group. We now have further appreciation of the complexity of the insect cuticle and clues about the need for so many CPR genes.

## Results and Discussion

### Overview

**Figure 2:**
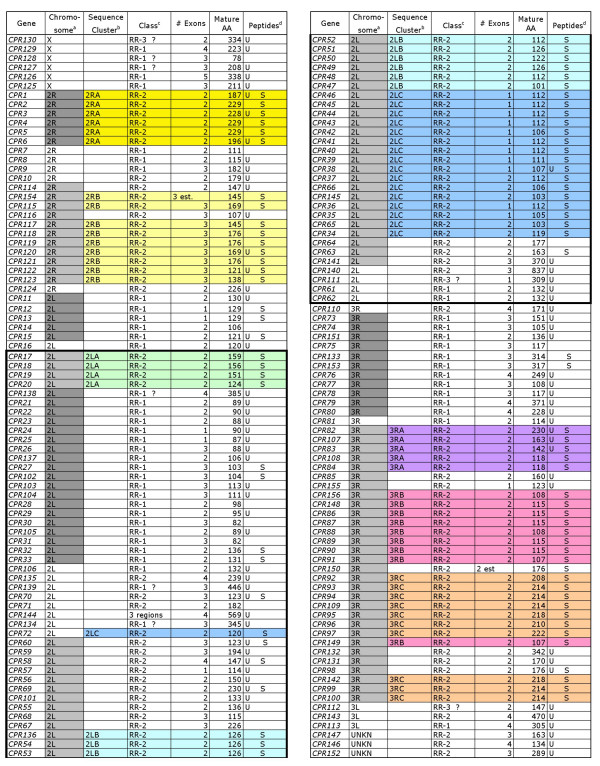
**Summary of *An. gambiae *genes/proteins with the R&R Consensus**. ^a^Tandem arrays are shown in alternating shades of gray. The sequences from *CPR17 *to *CPR62 *are boxed because they are included in the 2La inversion [29]. ^b^Sequence clusters are highlighted in color. ^c^Class for most sequences was determined using the tool at cuticleDB [17]. A ? indicates that manual assignment was necessary. ^d^Peptides indicates whether a unique peptide or a shared peptide was found for the corresponding protein via a proteomics analysis of cuticle preparations [12, He unpublished observations]. Proteins with data in this column are authentic not putative cuticular proteins. Additional details about the information in this Figure are in Additional Files 1, 2, 3, 4.

We have annotated 156 genes that have the potential to code for proteins with the R&R Consensus. RR-2 genes were 65% of the total. Genes are named in the order in which they were annotated. (See Methods for further details.) Their locations on chromosomes are given in Figure 2 and Additional Files 1, 2, 3, 4. We recognized eight tandem arrays (highlighted in gray) in which genes are typically spaced a few kb and never more than 20 kb apart. We also recognized eight sequence clusters, highlighted in different colors, and named by their order on chromosomes based on Ensembl genomic coordinates, e.g. 2LA, 2LB, and 2LC. Detailed information supporting each sequence cluster is provided in Cornman and Willis (MS in preparation). The same highlighting scheme is used for Figure 2 and on all relevant Tables.

### Validation of annotation

The 156 putative genes coding for proteins with the R&R Consensus is the largest group validated to date for any species. EST support from public sequence databases obtained at [18] had to be evaluated carefully, requiring matches in unique non-translated regions, because of the similarities within groups of genes. When this was done, a combination of available EST sequences and RACE and RT-PCR products sequenced by our laboratory confirmed 55% of the sequences (Additional File 3). A further 42% were confirmed as unique genes in the G3 strain by obtaining single-copy kinetics with qPCR on genomic DNA and confirming their expression with qRT-PCR (Additional File 3, [13]). Most primers for this analysis were designed around the stop codon to take advantage of the higher level of variation in the 3' UTR. Together these data uniquely confirm the large majority of annotated genes. Using the SignalP web tool [19], we also confirmed that each predicted protein had a valid signal peptide, an essential feature of secreted proteins. The presence of a TATA box and INR (initiator element) in 93% of the genes (see below) provided additional confirmation of the reality of these genes. Indeed, these features were important in several cases where Ensembl had predicted a single gene with multiple R&R Consensus regions, yet manual annotation revealed that multiple genes were present. A proteomics analysis based on head capsules and pupal cuticles left behind after a molt and some batch prepared cuticles [[12], He unpublished observations] revealed unique peptide(s) for 75 of these genes and shared peptides for an additional 70, having excluded those with only DGDVVK, a very common peptide. This information confirms 48% of genes we annotated as authentic components of the cuticle, and if we include proteins with shared peptides the proportion rises to 93% (Figure 2, Additional File 3). Finally, a combination of all of the supporting data has provided at least some support for all 156 CPR proteins (Additional File 3). Given that our experimental work was carried out on the G3 strain, not the PEST strain that was the source of the annotated genome, we were gratified by the support we found.

Did we identify all of the genes with the R&R Consensus? We only included three genes on unplaced contigs (i.e., on the ''UNKN'' chromosome). We had information about these three genes that supported their inclusion in our analysis. Fifteen of the 22 genes we ignored were on contigs less than 1 kb, and the longest was on a contig of only 2966 nucleotides. We assumed that these represent alternative haplotypes present in the PEST strain, which is reasonable given the level of haplotype variation that has been observed [11]. In one case, we omitted a tract of five candidate genes on chromosome 2R that are nearly identical to, but a subset of, another set of genes (*CPR115 – CPR123 *and *CPR154*) approximately 1 Mb away. The intergenic sequence in this tract was in some regions completely unresolved whereas in other regions it was virtually identical, preventing any direct verification of the tract by PCR. To ascertain whether all of these candidate genes were likely to be present in the PEST strain, we performed qPCR on genomic DNA with a primer set that included intronic sequences and was complementary to a subset of the candidate genes in question. We compared the data to results for a known single copy gene (chitin synthase, AGAP001748), which indicated that the actual number of targets was between five and six and not eight as expected if all of the candidate genes in the assembled genome were actually present (data not shown). This result was consistent with only the annotated genes being present in PEST; an identical result was also obtained with the G3 strain. The coordinates of the omitted sequences (labelled DUPL A-E) are provided in Additional File 1 for reference, but we believe there is insufficient evidence of their validity to warrant their inclusion in the present analysis. Thus, given the diverse data we used to annotate and confirm the CPR genes and our exhaustive BLAST searches, it is unlikely that many genes were missed or are artifacts of assembly or incorrect annotation. We also identified two pseudogenes as shown in Additional File 1, but we did not include them as named members of the gene family and they were excluded from analyses of molecular evolution. Seventy-six percent of the gene models are modifications or new genes relative to the Ensembl data available at the time the annotations were done. See Methods for details on retrieving all the sequences.

### Orthologs

Further verification of the annotation comes from orthologs in *D. melanogaster*. The data on orthologs are summarized in Additional File 5 where obvious differences between pairs are highlighted. Such comparisons reveal an important complication in the discovery of distant orthologs, namely that high-scoring reciprocal BLAST hits may be restricted to the R&R Consensus region with little conservation of the flanking sequence. This creates ambiguity as to whether a gene is a genuine ortholog or a product of parallel evolution or domain shuffling. We present data in Additional File 5 on sequence identity in both the Consensus and the total protein and have retained putative orthologs only in cases where the evidence was compelling or interesting.

We found good orthologs for seven of the 15 proteins that had over 300 amino acids in their mature form. This indicates that there is something important about their structure that has been preserved for about 250 myr [9].

There are instances where the regions flanking the Consensus are quite different in an ortholog pair, such as AgamCPR132 and DmelCry, a *D. melanogaster *lens protein [20]. The *An. gambiae *protein has a stretch of 20 glutamines in a row, while the *D. melanogaster *protein with almost the same percentage of glutamines has no cluster longer than 7. Given the propensity of glutamines to form amyloid-like structures, this suggests that the structure of the cuticle will be somewhat different in the two species. The length of the longest glutamine repeat was variable among the more than 50 cDNAs and ESTs that have been deposited in public databases from different strains (median = 23). Interestingly, while the long glutamine tracts in Huntington's and other human diseases are based on the expansion of just CAG repeats [21]; the long *An. gambiae *cluster uses both CAG and CAA codons. Cry along with AgamCPR132 had numerous repeats of RREE that may make a contribution to protein structure comparable to a stretch of glutamines.

Another example is illustrated by AgamCPR152 and the *D. melanogaster *protein resilin (CG15920). Resilin is a cuticular protein that confers elastic properties to specific regions of cuticle [22]. While the extended Consensus region of the two has 76% identity, the other regions of the protein are totally different. AgamCPR152 has 20% histidine residues, resilin had only 1 histidine. Dmelresilin is 35% glycine, AgamCPR152 only 12%. This is a clear case where the regions flanking the Consensus are not related in the two species. So is there a resilin gene in *Anopheles*? Lyons et al. [23] have made a recombinant protein with 16 units of a repeat (AQTPSSQYGAP) from an *An. gambiae *EST (BX61961) identified in a BLAST search using the *Drosophila *gene. When properly cross-linked, this protein has the same resilient elastic properties as a comparable multimer made with the *D. melanogaster *resilin repeat (GGRPSDSYGAPGGGN). The *An. gambiae *sequence corresponds to the incompletely annotated gene AGAP002367. This gene has 10 perfect copies of the repeat and three additional ones that differ in one amino acid. It lacks, however, an R&R Consensus even after probable errors in the original annotation were corrected and no Consensus region lurks in the surrounding 10 kb. This may well be a situation where domain shuffling has occurred.

Several other orthologs merit comment. While all other *An. gambiae *CPR proteins have only a single R&R Consensus region, there are three versions of it in AgamCPR144 and its orthologs DmelCpr73D and *Tribolium *GLEAN_16311. All three share other features. AgamCPR138 is a clear ortholog of the *D. melanogaster *protein l(3)mbn. The latter is longer, but they share two interesting features, the C-terminal position of the R&R Consensus and the presence of two cysteine residues that are rarely found in mature CPRs. We identified orthologs for two of the six *An. gambiae *genes that lacked the aromatic triad; one had a diad, the other only a single aromatic residue signifying the start of the R&R Consensus. Each had an ortholog with the same atypical feature.

Of the six CPR genes on the X chromosome, we identified orthologs for five, but only Agam*CPR129 *has an ortholog that also resides on the X chromosome in *D. melanogaster*. The other four orthologs are on 3R and three unrelated genes with the R&R Consensus are on the *D. melanogaster *X chromosome. It was apparent from this limited number of orthologs, that genes that are on either 2L or 3L in *An. gambiae *reside on 3L in *D. melanogaster*.

### Core promoter regions and polyA addition sites

For each gene, we summarized the presence of a TATA box, the position and sequence of its INR if one could be identified, and other features (Additional File 3). Analysis of the core promoter region was possible for 153 genes; the other three had their 5' region determined by RACE, since the genomic sequence was populated by a string of "Ns", an intra-contig gap, in the relevant regions. The vast majority, 126/153 (82%), of the CPR genes have a conventional TATA box (TATAAA). An additional 19 have a variant TATA box, including those in sequence cluster 2LC where 13/16 genes appeared to use TATTTAA. In all 145 cases where a TATA box was used, we were able to identify a putative INR. Cherbas and Cherbas [24] reported four common INRs in *D. melanogaster*, TCAGT, ACAGT, GCAGT, and TCATT. These sequences comprise 71% of the INRs we identified. Variants are shown in Additional File 3, some of which (italicized) were confirmed by 5' RACE, others were deduced by their distance downstream from the TATA box and the presence of an adenine in the third position. For seven of the eight genes with no known TATA box, we were able to identify INRs and putative DPEs (downstream promoter elements) [25]. The conventional polyA addition site (AATAAA) was found in 84% of the CPR genes within the first 500 nucleotides after the stop codon. Alternative sites (AATACA, AATATA, AATTAA) have been reported from some *Bombyx *cuticular protein genes [26,27]. We found the first two types in 14 additional genes. Hence all but 7% of the 153 genes that had sufficient C-terminal sequence data available had known polyA addition sites. The presence of appropriate untranslated flanking regions is further evidence for the authenticity of the genes we have annotated.

### Patterns of gene architecture

As is evident from Additional Files 1 and 2, *An. gambiae *CPR genes show considerable variation in exon position and length. Complete coding sequences from *genomic *DNA are obtainable for 154 of these proteins; two others, with "Ns" in the genomic data, were obtained by sequencing RACE products. Eighteen sequences had only a single exon, 11 of which were in sequence cluster 2LC; one sequence had five exons. The majority (55%) had two exons, with three and four exons present in 25%, and 8%, respectively. A large majority (86%) of genes with introns had an interruption in the region coding for the signal peptide, resulting in short first exons of from 3–36 nucleotides long, excluding the 5'UTR. Both the median and mode were 12. Short first introns are not unique to CPR genes but are an obstacle to gene annotation. This is because several plausible first exons may fit a well-supported gene model and because EST support for first exons is sometimes lacking. We cloned and sequenced RACE products from the G3 strain to verify some problematic first exons (see Additional File 3), but we did not do so routinely for gene models that were well supported by similarity to related CPR genes.

Intron number is variable throughout the CPR phylogeny, suggesting that intron loss or gain has occurred repeatedly. Overall, however, there are general patterns that distinguish the exon structure of RR-1 and RR-2 genes. There are fewer RR-2 genes with more than two introns than RR-1 genes (23% vs. 52%), and exon-intron boundaries are more strongly biased toward phase 0 (i.e. between codons) in RR-2 genes (96% of introns) than in RR-1 genes (63%). Furthermore, the interruption of the Consensus region by an intron is less common in RR-2 genes (15%) than in RR-1 genes (49%). Indels are very rare in the aligned RR-2 Consensus region and span at most two codons, whereas the aligned RR-1 Consensus region has extensive indels, particularly near the center of the alignment. Thus, the two classes differ substantially in the structural diversity of the R&R Consensus. These architectural differences also suggest that RR-2 proteins may be more amenable to novel gene formation by transposition of an intact R&R Consensus domain, although such an occurrence has not been demonstrated for a CPR gene.

### Strain variation in gene number

**Figure 3:**
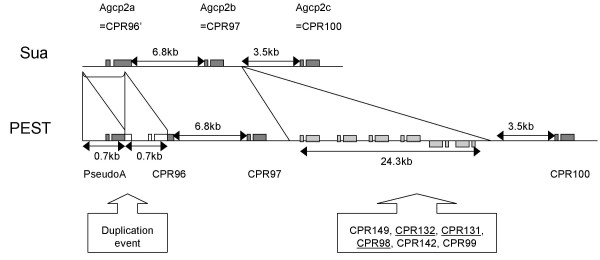
**Example of strain variation in gene number and/or order within *An. gambiae***. Orthologous genomic regions from the Sua and PEST strains that contain different numbers of CPR genes are depicted. The Sua clone sequenced by Dotson et al. [14] contains three CPR genes, two of which, *Agcp2b *and *Agcp2c*, are orthologs of the genes *CPR97 *and *CPR100 *that we identified in the PEST strain. Due to a duplication event, the other Sua gene, *Agcp2a*, is orthologous to both the first half of the pseudogene *PseudoA *and the second half of *CPR96*. The intergenic regions between the three Sua genes are also present in the PEST genome sequence, but there is also an additional 24.3 kb segment containing six additional CPR genes. It is not known whether this segment is absent in the Sua strain or has simply been rearranged relative to PEST. Diagram is not drawn to scale; single-copy genes (those not in sequence clusters) are underlined.

While there has been no systematic survey of variation in CPR gene number within a species, serendipitous findings of gene copy number variation suggest that it may be common, particularly in large tandem arrays. For example, investigators have identified strain variation in CPR gene number within *D. melanogaster *[10,28]. In the present study, we identified a large tandem array of RR-2 genes on 3R that appears to be particularly dynamic with respect to gene copy number. Previously, Dotson et al. [14] had sequenced a 17.4 kb genomic clone from the Sua strain of *An. gambiae *and identified 3 CPR genes (*Agcp2a,b,c*) in this genomic region. We were able to identify orthologs for all three genes in the PEST strain, but *Agcp2b *(*CPR97*) is separated from *2c *(*CPR100*) by 24.3 kb rather than 3.5 kb (Figure 3, Additional File 1). That larger region has six additional CPR genes. Two are in the same sequence cluster, one belongs to another sequence cluster, and three are single-copy genes. We have not investigated if the additional region or its component genes are present somewhere else in the Sua strain. Whether copy number variation is prevalent or adaptive in natural populations remains to be determined.

An inversion on chromosome 2L in some strains of *An. gambiae *is of particular interest because one form (2La) correlates with desiccation resistance and vector competence discussed in Sharakhov et al. [29]. Their careful mapping of the inversion boundaries enabled us to place it in the context of the genes we have annotated with the surprising result that there are 73 CPR genes within the inverted region (Figure 2, Additional File 1). We used inversion specific primers [30] to verify that the G3 strain, which we used for analysis, is heterozygous for the inversion (data not shown).

### General protein properties

A summary of protein properties is in Table 1 and Additional File 4. The CPR proteins averaged 169 residues (minus the signal peptide), with a considerable range, 87–837. The major feature of the CPR proteins is, of course, the presence of the R&R Consensus (see Figure 1). In all but six sequences, it began with the aromatic triad described above; in four cases only a diad was found, and in two cases a single aromatic residue defined the start of the Consensus region. The position of the start of the R&R Consensus ranges from the fourth to the 90^th ^percentile of the length of the mature protein, and the average position was just at the start of the second quartile (27^th ^percentile). Glycine and alanine are major amino acids; 26 proteins have over one fourth of their residues as just these two amino acids. Three RR-1 proteins (CPR79, CPR133, and CPR153) had over 40% of their residues as a combination of just these two amino acids. Proline is another major amino acid in many of the proteins, reaching 15% in CPR79; the average was 7%. Both proline and glycine-glycine pairs tend to introduce kinks in protein chains and thereby influence protein chain folding [2]. Both histidine and lysine have been shown to be involved in sclerotization, the cross-linking of quinone derivatives to the proteins [31]. On average, RR-2 genes have more histidine residues, 11% vs. 2% (Table 1, Additional File 4).

**Table 1:** Properties of CPRs by class and by individual RR-2 sequence clusters

CLASS	N	Avg. AA	SIGNAL	% to START of CONSENSUS	% HIS	% LYS	% PRO	% GLY	% ALA	% G+A
RR-1	54	160	18	27	2.2	3.7	8	9.8	9.2	19
RR-2	101	167	18	27	11*	5.6	6.4	8.7	10.9	20
										
2RA	6	216	17	24	1.7	5.2	9.5	3.3	25*	28*
2RB	9	157	17	35	2.8	4.3	11	7.9	25*	28*
2LA	4	148	17	38	4.6	9.7	2.1	26*	2.1	28*
2LB	9	120	17	16	17*	8.3	6	10	5.8	16
2LC	16	110	17	12	17*	6.5	2.7	13	6	19
3RA	5	154	17	21	7.2	6.5	7.8	3.9	13	17
3RB	9	112	17	24	19*	4.8	5.2	8.2	4.6	13
3RC	10	215	17	33	18*	2.3	7.5	4.1	17	21

Distinguishing features are followed by a *.

**Figure 4:**
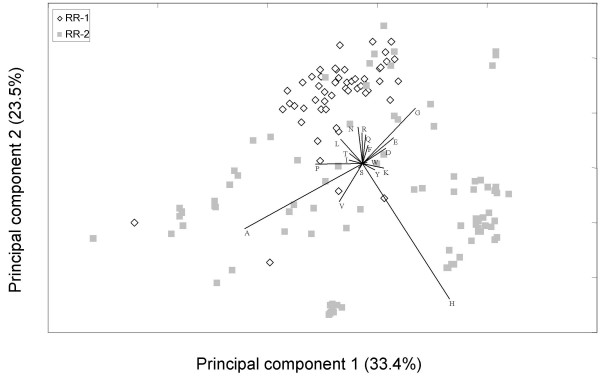
**Scatterplot representing variation in amino-acid content of all putative *An. gambiae *CPR proteins**. The horizontal and vertical axes are the first and second principal components, respectively, of the amino-acid composition matrix after removal of the predicted signal peptide. The labeled vectors indicate the relative loadings of each amino acid in the first and second principal components. The amino acids cysteine and methionine, which are scarce in mature CPR proteins (0.03 and 0.4% respectively), were excluded from the composition matrix. The percentage of the total variation explained by each axis is also given. RR-1 and RR-2 proteins are indicated separately according to the legend. PC axis 1 and 2 = 33.4 + 23.5 = 57% of total variation. The major loadings for the first five PC axes (82% of the variation) are: A, H, G-Q, V, Q-Y. RR-1 and RR-2 are strongly separated on the second axis and RR-2s have more variation overall.

We used principal components analysis (PCA) of the amino-acid composition matrix to further investigate patterns of variation in amino-acid content of RR-1 and RR-2 proteins. For this analysis, we excluded cysteine and methionine because these two amino acids are virtually absent from mature CPR proteins (Additional File 4), hence their removal reduces the dimensionality and noise of the composition matrix. Figure 4 shows a scatterplot of all CPR proteins along the two major principal component axes, which together explain 57% of the total variation. The labelled vectors represent the relative contribution of each amino acid to the variation explained by these two axes. Not surprising given the composition values mentioned above, alanine, histidine, and glycine are prominent in discriminating among proteins. Two features of CPR diversity are particularly apparent from Figure 4. First, RR-1 proteins show much less variation in amino-acid composition than RR-2 proteins. Secondly, RR-1 and RR-2 proteins are strongly discriminated by the second principal-component axis, for which the proportion of histidine is the most strongly loaded variable.

### Commonly occurring sequence motifs

Within the two major classes of the R&R Consensus sequence, some common variants are recognizable that extend the region of alignable sequence in the N- or C-terminal directions for a subset of genes. One common variant found in RR-1 genes has a proline-rich region adjacent to the defined Consensus (Figure 1), which can be approximated by the expression GFQPQGxHxPxPPP. A second common variant is found in RR-2 genes at the C-terminus of the R&R Consensus, approximated by the expression GFNAVV(HR)RE(GP). Also present in 38 RR-2 genes was RDGDVVKG. All three of these variants occur in tandem gene arrays in *An. gambiae *as well as other Dipterans, indicating that they arose to high frequency by tandem gene duplication (Cornman and Willis, MS in preparation). The first two, however, are also present in *Apis *and *Tribolium *(Cornman, unpublished data) indicating that these motifs are old and, given their high level of conservation, probably have functional importance, possibly in addition to their participation in chitin-binding.

**Figure 5:**
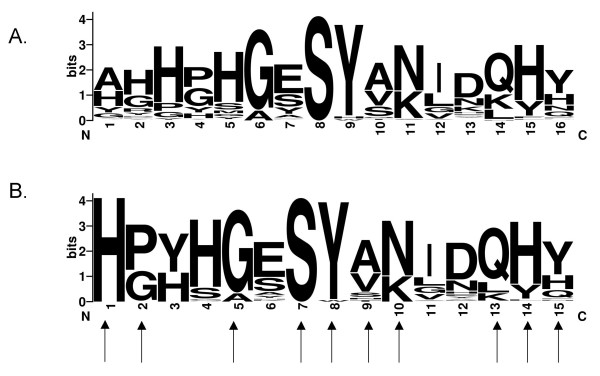
**C-terminal sequence motif identified by MEME analysis**. A. Sequence logo representing actual matches to the motif position-specific scoring matrix in *An. gambiae *and *Ae. aegypti*. The motif is present in 29 *An. gambiae *RR-2 genes on chromosome 2L and in 65 annotated genes in *Ae. aegypti*. B. Sequence logo representing a modified version of the motif that is reduced by one position and permits indels. Arrows indicate sites under negative selection at P < 0.05, as determined by single-likelihood-ancestor counting [32]. Overall Ka/Ks for the modified motif was estimated to be 0.42 [95% CI: 0.39, 0.65].

CPR genes frequently contain low complexity sequence in the regions flanking the R&R Consensus, and several common repeats have been recognized (reviewed in [2,3]). Andersen et al. [2] described a common repeat in cuticular proteins, AAP(A/V). We identified this repeat or a variant form, AAPL, in one quarter of *An. gambiae *CPR proteins (Additional File 4). To systematically search for more complex sequence patterns outside of the R&R Consensus, we used the MEME server (see Methods). For this analysis, we removed the signal peptide and R&R Consensus so that they would not influence the motif search. We submitted all annotated *An. gambiae *and *Aedes aegypti *CPR genes to achieve the best representation of sequence variation, and we ensured that no putative motif spanned the artificial gap created by removing the Consensus. To eliminate the effect of duplication *per se*, we ignored any motifs present only within a single sequence cluster. Given the high proportion of low-complexity sequence, we also required that the motif be more than five amino-acids long. Only one motif was found that met these criteria, a 16 amino-acid motif that is present at or very near the C-terminus of all sequences that contain it. All *An. gambiae *genes with this motif are RR-2 genes that occur on chromosome 2L. A sequence logo representation of observed matches is shown in Figure 5A. The position-specific scoring matrix for this motif, which can be used to search custom datasets via the MAST option of the MEME server, is presented as Additional File 6. When we compared the motif matches to the full alignment of protein sequences, it was apparent that a more concise motif could be obtained (Figure 5B) by removing the first position and allowing single-position indels. It is interesting to note that this motif is rich in histidine, which is along with alanine and glycine a major contributor to variation in amino-acid composition among CPR proteins as discussed above. We show in a later section that this modified motif is under purifying selection.

### Phylogeny and genomic organization of CPR genes

In *An. gambiae*, CPR proteins cannot generally be aligned outside of the R&R Consensus and signal peptide, consistent with the pattern of insect CPRs generally. We therefore based our phylogenetic analysis on the region that spans the R&R Consensus from the fifth position N-terminal to the tenth position C-terminal of the pfam00379 sequence (Figure 1). We included these flanking regions because, while they are not alignable across all *An. gambiae *CPRs, they incorporate common sequence variants that are alignable within large subsets of genes and thus are phylogenetically informative. However, we double-weighted all positions from the aromatic triad to two positions past the final invariant glycine (Figure 1) as these positions can be aligned across all CPRs. We used amino-acid sequence rather than codon-aligned nucleotide sequence because mutational saturation of the latter is evident across the gene family as a whole.

**Figure 6:**
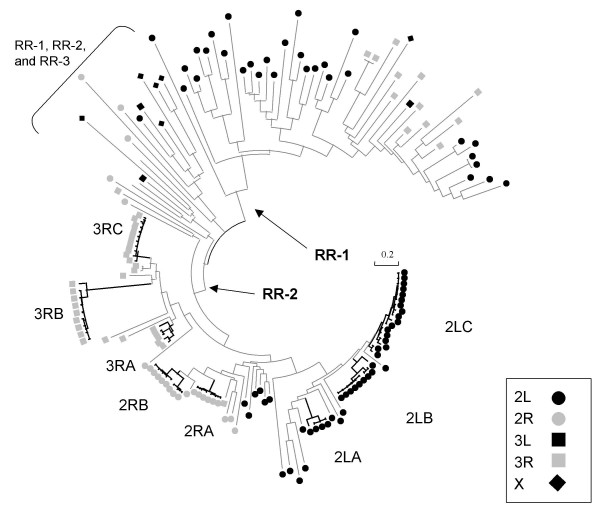
**Topology of the neighbor-joining tree of all *An. gambiae *CPR genes**. Gene names are omitted here for clarity. Detailed phylogenies of the RR-1 and RR-2 clades are presented as Figures 7 and 8. Distances are based on the amino-acid sequence of the aligned and weighted R&R Consensus as described in the text. We used the JTT exchange matrix [49] as implemented in MEGA3 [48]. The core group of RR-1 and RR-2 proteins and an intermediate group of RR-1, RR-2, and RR-3 proteins are marked. The sequence clusters defined in the text are also marked. Symbols represent the chromosome arm on which each gene is located. The three without symbols have not been placed on a chromosome.

**Figure 7:**
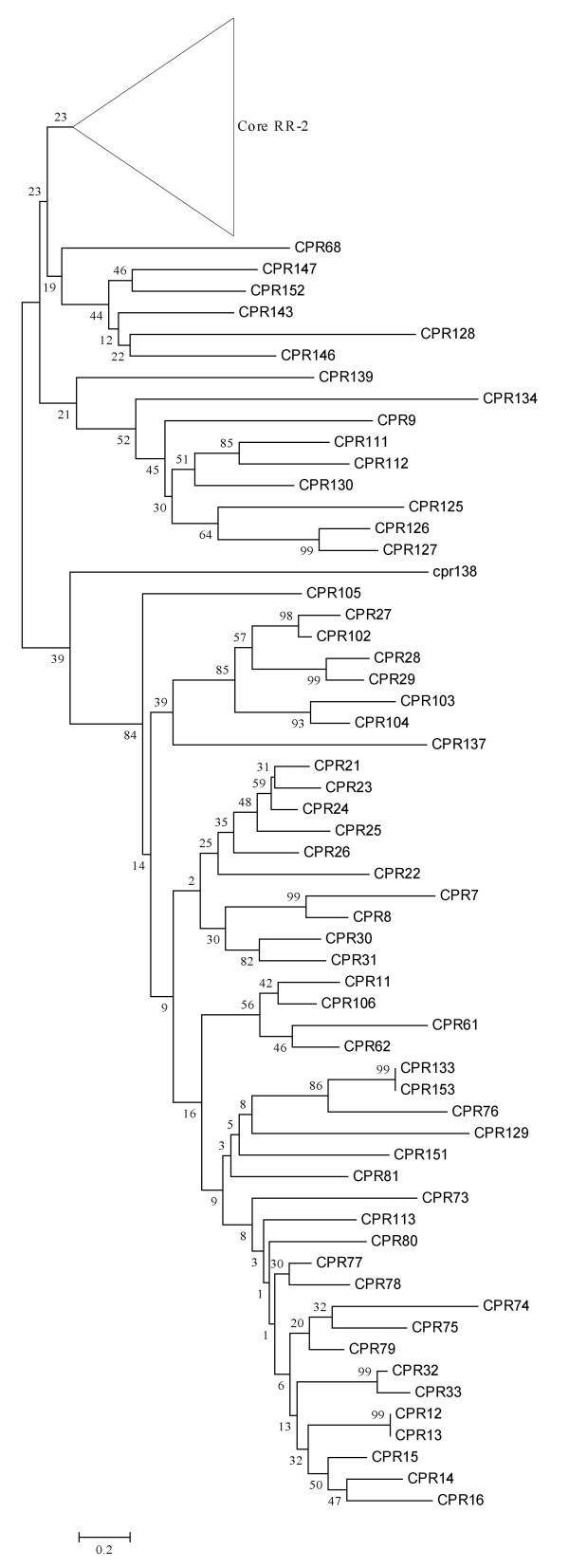
**Phylogenetic relationships of the core RR-1 and intermediate CPR proteins**. A detailed view of the neighbor-joining tree of *An. gambiae *CPR genes with the branch leading to the main group of RR-2 proteins shown in Figure 6 collapsed. Numbers at nodes indicate the percentage of 1000 bootstrap replicates that support the node.

**Figure 8:**
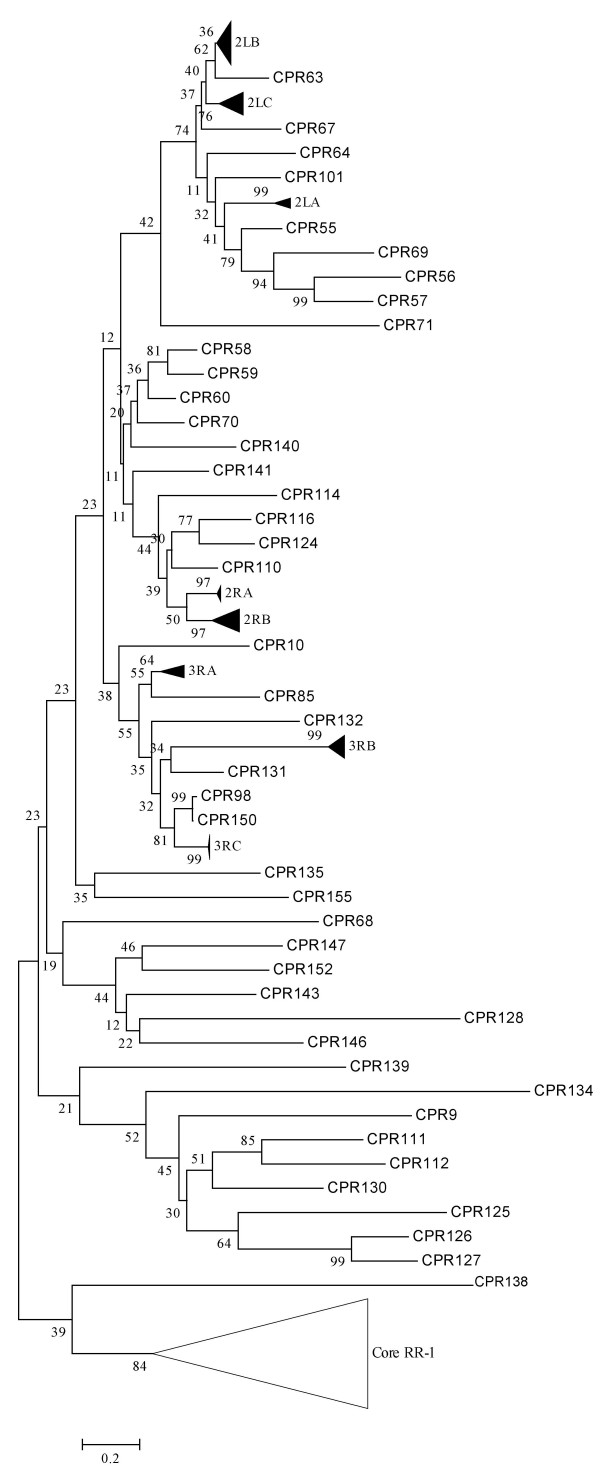
**Phylogenetic relationships of the core RR-2 proteins**. A detailed view of the neighbor-joining tree of *An. gambiae *CPR genes with the branch leading to the main group of RR-1 proteins shown in Figure 6 collapsed as are the individual sequence clusters. Numbers at nodes indicate the percentage of 1000 bootstrap replicates that support the node.

The neighbor-joining phylogeny we recovered identifies two main groups that constitute the core RR-1 and RR-2 proteins. Figure 6 shows the overall topology of the tree; detailed phylogenies of RR-1 and RR-2 genes are presented as Figures 7 and 8. The RR-2 clade has much shorter branch lengths on average than the RR-1 clade because the former group is dominated by sequence clusters. Several long-branch genes lie between the main RR-1 and RR-2 clades. This group has low bootstrap support and is probably an artificial group caused by long-branch attraction. Both RR-1 and RR-2 genes are present in this group. The group includes five of the six X-chromosome genes as well as all three genes that match the "RR-3" designation of Andersen et al. [16] which are indicated in Figure 6. Removal of this group of long-branch genes raises bootstrap support for the core RR-1 and RR-2 clades to 100% (results not shown).

CPR genes are found on all of the chromosomal arms except for the Y chromosome but have a biased distribution with 51% occurring on 2L and 28% occurring on 3R. Phylogenetically, genes show a very strong tendency to cluster by chromosome (Figure 2, Figure 6), indicating that inter-chromosome duplications are rare. RR-1 and RR-2 tandem arrays show no overlap on chromosomes (Figure 2, Additional File 1) and contain few non-CPR genes.

### Evolutionary patterns within the R&R Consensus and flanking sequence

The pattern of natural selection acting during the evolutionary history of a gene family can be illuminated by comparing the rate of nucleotide substitutions that change the protein sequence (Ka) to the rate of nucleotide substitutions that do not (Ks). Coding sequences that have a ratio of Ka to Ks equal to one are evolving in a manner consistent with neutrality, that is, nonsynonymous mutations are as likely to be fixed as synonymous ones. Ka/Ks greater than one indicates a higher rate of amino-acid substitution than expected under neutrality and implies that positive selection has driven the divergence from the ancestral state. Ka/Ks lower than one implies that changes in protein sequence are selected against on average.

To investigate the possibility of adaptive evolution of the R&R Consensus during the diversification of CPR genes, we calculated Ka/Ks within this region for all *An. gambiae *paralog pairs and *An. gambiae *– *Ae. aegypti *ortholog pairs that we identified. For this analysis, we used a shortened version of pfam00379, in which we excluded the first seven positions, to define a more strict R&R Consensus for *An. gambiae*. This was done because the alignment of the first seven sites across all RR-1 or RR-2 proteins was ambiguous and therefore not useful for analyses of substitution patterns.

One difficulty with the interpretation of Ka/Ks ratios between functional paralogs is that a finding of increased Ka/Ks after duplication may be explained by relaxed selection on initially redundant genes or by adaptive evolution at some fraction of sites within a functionally constrained sequence, or both successively. Only if Ka/Ks is substantially greater than one is adaptive evolution strongly implicated, although it remains a possible explanation for even small increases in Ka/Ks. Codon-based models of nucleotide substitution are potentially more useful because they can identify individual sites under selection within a local region of low Ka/Ks, but their power is strongly dependent on sample size [32] and the actual pattern of selection. We used both approaches to investigate the molecular evolution of the chitin-binding R&R Consensus, which has a well-characterized secondary structure and both labile and highly conserved positions [8,33].

**Figure 9:**
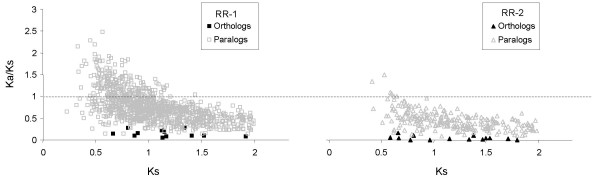
**Ka/Ks of RR-1 and RR-2 single-copy genes**. Ka/Ks is plotted as a function of Ks for all *An. gambiae *paralog pairs and all *An. gambiae *– *Ae. aegypti *ortholog pairs for which the estimated Ks < 2. Genes in sequence clusters are excluded because they are likely to be products of recent duplication or gene conversion, which can bias Ka/Ks.

Figure 9 shows mean Ka/Ks for all pairwise comparisons among single-copy RR-1 and RR-2 paralogs, respectively, for which the Jukes-Cantor corrected Ks is less than 2 (to reduce the effect of mutational saturation). The Ka/Ks between ortholog pairs in *An. gambiae *and *Ae. aegypti *with corrected Ks < 2 are also shown for comparison. Ka/Ks is higher on average among RR-1 paralogs than RR-2. A significant fraction of RR-1 gene pairs have Ka/Ks greater than 1, whereas RR-2 genes pairs have Ka/Ks almost exclusively below 1. Interestingly, the six CPR genes on the X chromosome (*CPR125 *– *CPR130*) were all among the genes with the highest average pairwise Ka/Ks (Table 2). This finding accords with previous work [34] that found a significant increase in the Ka/Ks of X-linked versus autosomal duplicates in *D. melanogaster*. The higher rate of evolution of X-linked duplicates is a theoretical prediction if one assumes that most advantageous mutations are recessive and hence more visible to selection when X-linked [35].

**Table 2:** CPR genes with the highest mean pairwise Ka/Ks. Genes in bold are on the X chromosome

Gene	Ka/Ks
*CPR9*	1.12
***CPR130***	1.08
***CPR128***	1.01
***CPR125***	0.99
***CPR126***	0.97
*CPR134*	0.96
*CPR111*	0.95
***CPR127***	0.91
*CPR16*	0.89
***CPR129***	0.87

We next used codon- and branch-based tests of selection to test for positive selection on the R&R Consensus of *An. gambiae *CPRs. We used the single-likely-ancestor counting method, SLAC [32], and GA-Branch method [36] implemented by the DataMonkey web server [37]. The SLAC method identifies individual codons that deviate from neutral expectation with respect to nonsynonymous versus synonymous substitutions, whereas the GA-Branch method identifies lineages of a phylogeny that differ in overall Ka/Ks. Subsets of CPR genes were investigated by submitting well supported interior clades that are likely to have experienced less mutational saturation and by removing highly similar gene duplicates. In no case did we find any positively selected sites within the R&R Consensus at P < 0.05, whereas a large majority of sites were found to be under negative selection. Conceptually similar but methodologically distinct methods for identifying sites under positive selection were also implemented with the codeml program of the PAML package [38] and gave similar results. Specifically, evolutionary models that included a category of Ka/Ks > 1 in addition to either a single category of Ka/Ks < 1 or a beta distribution of Ka/Ks < 1 were not significantly more likely than models that did not include positive selection (codeml model options 2 versus 1 and options 8 versus 7, respectively, 2ΔlnL ≈ 0 for both comparisons). Thus, diversification of the CPR gene family does not appear to have been driven by strong positive selection on specific sites within the R&R Consensus. We estimated the global branch Ka/Ks within the R&R Consensus under the beta-distribution model (model 7) of codeml, which was significantly more likely than a single Ka/Ks < 1 category (model 1) for both the RR-1 and RR-2 clades (P << 0.01), and after removing all but one haphazardly chosen gene of each sequence cluster. The evolutionary rate of amino-acid substitutions was more than twofold higher within the RR-1 clade (Ka/Ks = 0.271) than within the RR-2 clade (Ka/Ks = 0.118).

Although no sites or sites within lineages showed significant evidence of positive selection, the relative rates of amino-acid evolution are nonetheless variable among lineages, as indicated by Tajima's test [39]. The most notable example is the branch leading to the 3RB sequence cluster (Figures 6, 8) compared with the linked sequence clusters 3RA and 3RC (average P < 0.01 across all possible gene comparisons for the three sequence clusters). Since we found no support for elevated Ka/Ks occurring along this or any other lineage as described above, the high rate of evolution in the 3RB sequence cluster appears to be due to mutation-rate variation alone. Consistent with this interpretation, the 3RB sequence cluster has a substantially lower GC content (36.9%) than the other 3R sequence clusters (46.7%), suggesting divergent mutational histories. This localized difference in GC content is surprising given the very tight linkage of the genes in these three sequence clusters (Additional File 1).

We also performed a SLAC analysis on all examples of the motif identified by MEME (aligned with indels) from *An. gambiae *and *Ae. aegypti*. Ten of fifteen sites were found to be under purifying selection at P < 0.05, as shown in Figure 5B, and the Ka/Ks for the region was estimated to be 0.42 (95% C.I. 0.39, 0.65). While the motif is evidence of sequence conservation among paralogs outside the R&R Consensus, the level of conservation is somewhat less than what is seen among single-copy orthologs in the two mosquito genomes (Ka/Ks of 0.08 – 0.33 with the R&R Consensus removed).

Our analysis of the CPR gene family in *An. gambiae *reveals that a high number of paralogs can be retained in insect genomes with remarkably low rates of pseudogene formation. The retention of these paralogous single-copy genes or sequence clusters over periods of 100 million years or more, as evidenced by orthologs in *Ae. aegypti *(Cornman and Willis, MS in preparation), implies that there has been extensive functional diversification of this group. We do not yet know the nature of this functional diversification, although there is certainly broad variation in the complement of CPR proteins expressed at different developmental stages and in different tissues as evidenced by gene expression and proteomic studies ([12,13], He unpublished data). There are also a few examples from *D. melanogaster *of CPR proteins that have been shown to be essential components of specialized cuticular structures, such as crystallin in the eyes [22] and resilin in wing tendons, ligaments, etc. [22,23]. Nonetheless, codon-based analyses of the R&R Consensus of *An. gambiae *CPR proteins failed to identify any sites under positive selection, although such tests can have limited power to detect ancient positive selection and Ka/Ks>1 within gene families is rarely found [40].

Our comparison of the RR-1 and RR-2 classes presents an interesting dichotomy regarding the diversification of the R&R Consensus and flanking sequences. The R&R Consensus of the RR-1 class has higher rates of amino-acid evolution and is structurally much more variable than the RR-2 family, and a number of RR-1 proteins have average pairwise Ka/Ks approaching one, many times higher than the mean Ka/Ks for orthologous pairs in *An. gambiae *and *Ae. aegypti*. To the extent that there has been adaptive evolution of the chitin-binding Consensus, it has probably been more prevalent in the RR-1 clade. In contrast, the RR-2 clade appears to have diversified in terms of amino-acid composition to a much greater extent than the RR-1 class and in general there is a higher frequency of histidine residues, which are involved in cross-linking, in this group. It seems likely that the functional diversification of the RR-2 group derives to a greater extent from the properties of the sequence flanking the Consensus than from any particular changes in the chitin-binding sequence itself.

One of the most remarkable features of this gene family in *An. gambiae*, the presence of sequence clusters of highly similar genes, begs investigation as to the possible selective advantage of increased copy number for these particular genes. A separate analysis of these genes was too extensive to include here, but our unpublished data indicate that these proteins have complex repeats and unusual amino-acid compositions relative even to other RR-2 proteins. Furthermore, different sequence clusters appear to have independently acquired compact gene architectures, perhaps as a response to selection for increased gene expression, and concerted evolution in addition to purifying selection appears to be an important mode by which protein similarity is maintained in these groups.

## Conclusion

We have identified 156 genes containing the R&R Consensus in the genome sequence of An. gambiae; many have been confirmed experimentally. Phylogenetic analysis of these genes reveals three broad groups, the core RR-1 and RR-2 groups plus an intermediate, long-branched group that is probably artificial. The RR-2 class is dominated by sets of highly similar genes that we have termed sequence clusters. The RR-1 class of the R&R Consensus has a higher evolutionary rate than the RR-2 class, whereas the latter group has a greater diversity of amino-acid composition in the flanking sequences. The multiplicity of almost identical genes within sequences clusters suggests that their amplification may serve to allow massive protein synthesis during the brief periods of cuticle secretion. In addition, and perhaps most importantly, differences among sequence clusters, the rarity of pseudogenes, and the presence of good orthologs for several single-copy genes all indicate that the distinct CPR proteins serve important and unique roles in the cuticle.

## Methods

### Annotation of sequences

Prior to the availability of sequences from completely sequenced insect genomes, there were 98 insect cuticular proteins sequences available that had the R&R Consensus. Where corresponding genes were known, they were generally simple, with rarely more than two exons. The first intron frequently interrupted the region coding for the signal peptide. The second exon also began in a relatively conserved position close to or interrupting the nucleotides that coded for an aromatic triad near the N-terminus of the extended Consensus [3]. This triad is Y/F-x-Y/F/W-x-Y/F. These simple and consistent features guided the annotation of the *An. gambiae *genome. We also used the information from [14] that genes that coded for nearly identical proteins would differ in their 5' and 3' UTRs.

We investigated all gene predictions that contained the pfam00379 motif by searching the Ensembl *Anopheles *Web Site [41] with IPR000618 Additional sequences were identified when our proteomics project [12] turned up peptides indicative of belonging to a previously unannotated protein with the R&R Consensus. Additional BLAST searches and dot-matrix plots were also employed to confirm that we had identified all genes.

We examined each candidate gene to identify essential or common gene features, namely a TATA box, INR [24], signal peptide (identified by SignalP [19,42], R&R Consensus sequence, and poly-adenylation site (AATAAA or rarely AATACA). These were manually identified, guided whenever possible by ESTs available via BLAST searching [18]. The program Splice Predictor [43] was used to guide identification of splice sites. In a few cases, putative orthologs in other species provided valuable clues. For a few of the more difficult sequences, we used 5' or 3' RACE or RT-PCR to verify/complete the sequence. (See Additional File 7 for primers used). Such experimental work was, of necessity, done with the G3 strain as the PEST strain used for the whole genome sequencing no longer exists. Once sequences were annotated we used SPIDEY [44] to locate the coordinates on the appropriate contig. We present only positions that correspond to the coding sequence itself.

A summary of all annotated genes, their properties, and the nature of supporting evidence is given in Figure 2 and Additional Files 1, 2, 3. Genes in these tables are ordered by their appearance on chromosome arms. We were advised not to use chromosome bands for names as had been done for some of the *D. melanogaster *genes coding for cuticular proteins [10], so we settled for naming them simply as *CPR#*. When the genes are discussed in the context of genes in other species, their complete name should be *AgamCPR#*. Genes were named in the order in which they were annotated; so many names were added out of numerical order. We have provided the VectorBase stable identifiers that begin with AGAP in Additional File 1. A few genes that are incorrect are noted. *CPR150–156 *were not available when the naming occurred and no Ensembl gene names exist for these.

Definitive gene names, sequences (both cDNA and protein), plus contig locations were submitted to Ensembl and can be found at [45]. Protein sequences are also available at cuticleDB [17,46].

### cDNA cloning

For genes with ambiguous predictions, RT-PCR or RACE (rapid amplification of cDNA ends) was performed. RNA isolation and RT-PCR procedures were described before [13,47]. All RACE products except for 3' RACE of *CPR140 *and *CPR152 *were obtained with the GeneRacer^® ^kit (Invitrogen) using their SuperScript III reverse transcriptase. The 5' RACE product should reach the transcription start site. All PCR products were amplified with tag primers and/or gene specific primers listed in Additional File 7 by LA Taq or Ex Taq (TaKaRa) and cloned in pGEM-T Easy (Promega) or pCR4-TOPO (Invitrogen) plasmid vectors. For *CPR140 *and *CPR152 *3' RACE, first strand cDNA was synthesized by SuperScript III reverse transcriptase (Invitrogen) with an oligo dT-anchor primer (5'-GACCACGCGTATCGATGTCGACT_23_-3'), a modified version of the primer from the Roche RACE kit. A PCR anchor primer (5'-GACCACGCGTATCGATGTCGAC-3') and gene-specific primers were used with these two cDNAs. These modifications were necessary because the 3' RACE primers provided by Invitrogen frequently amplified incorrect regions of the *An. gambiae *genome by priming at both ends of a sequence. All other gene-specific primers used are given in Additional File 7.

### Phylogenetics and sequence analysis

The R&R Consensus was aligned with ClustalW using gap penalties of 10 to open and 5 to extend. The alignment was then manually adjusted to ensure consistency with pfam00379, particularly at the edges of the alignment, and to ensure that alignments among sets of highly similar genes were not distorted by the global optimum. The MEGA3 program [48] was used to calculate genetic distances using the JTT exchangeability matrix [49]. The distance measure is the expected fraction of accepted mutations scaled such that a distance of 1.0 is equivalent to 100 iterations of the JTT matrix. All distances were computed with pairwise deletion of indels.

All annotated *An. gambiae *CPR genes and all annotated *Ae. aegypti *RR-2 genes listed in Cornman and Willis (MS in preparation) were submitted to the MEME server [50,51] with the extended R&R Consensus and signal peptide removed. We placed no restriction on the number of motifs per sequence but limited the size to 5 – 25 amino acids per motif. We investigated the stability of the MEME-identified motifs by submitting haphazard subsamples of CPR genes from mosquito as well as other insect CPR genes. This was to ensure that the same motif definition was recovered when background sequence probabilities were modulated in this way.

The model of nucleotide evolution for SLAC analysis was chosen independently for each run using the DataMonkey model-selection tool [37]. Selected codons were assessed using a Bonferroni-corrected alpha of 0.05. Pairwise Ka/Ks ratios were calculated using the method of Nei and Gojobori [52] as implemented by the program DnaSP [53]. The program codeml of the PAML package [38] was used to assess the likelihood of sequential pairs of evolutionary models described by Yang et al. [54]. For each pair of models, the model with the fewest parameters was considered the null hypothesis and was rejected if the alternative model had a significantly higher likelihood by the chi-squared test suggested by Yang et al. [54].

## List of abbreviations used

CPR, cuticular protein(s) with the R&R Consensus; DPE, downstream promoter element; EST, expressed sequence tag; INR, initiator element; Ka/Ks, ratio of nonsynonymous to synonymous substitutions; PCA, principal components analysis; qPCR, quantitative polymerase chain reaction with DNA template; qRT-PCR, quantitative reverse transcriptase PCR; RACE, rapid amplification of cDNA ends; R&R Consensus, Rebers and Riddiford Consensus; RR-1, RR-2, two classes of CPR proteins; UTR, untranslated region of an mRNA; SLAC, single-likely-ancestor counting method.

## Authors' contributions

All of the authors contributed to the annotation. RSC and JHW wrote the manuscript; TT directed the experimental work on gene verification aided by ACE; RSC carried out the phylogenetic and other sequence analyses; WAD implemented data management. NH contributed unpublished data on proteomics. The project was conceived and coordinated by JHW. All authors have read and approved the final version of this manuscript.

## Supplementary Material

Additional file 1 Supplementary Table 1. Position of CPR genes (coding region only) on contigs arranged in their order on chromosomes. Click here for file

Additional file 2 Supplementary Table 2. General information about CPR genes. Click here for file

Additional file 3 Supplementary Table 3. Evidence that supports annotation. Click here for file

Additional file 4 Supplementary Table 4. Selected features of CPR proteins. Click here for file

Additional file 5 Supplementary Table 5. Comparison of *An. gambiae *and *D. melanogaster *orthologs. Click here for file

Additional file 6 Supplementary Table 6. Position-specific scoring matrix of the motif identified at the C-terminus of most RR-2 genes on chromosome 2L. Click here for file

Additional file 7 Supplementary Table 7. Oligonucleotide primers used for obtaining RACE products and RT-PCR products from selected genes. Click here for file
