# Supplementary material for: Annotation and analysis of a large cuticular protein family with the R&R Consensus in Anopheles gambiae
Source: BMC Genomics. 2008 Jan 18;9:22. doi: 10.1186/1471-2164-9-22 (PMC2259329; doi:10.1186/1471-2164-9-22)
Supplement: Additional file 1 — Supplementary Table 1. Position of CPR genes (coding region only) on contigs arranged in their order on chromosomes. [file 1471-2164-9-22-S1.PDF]

Supplementary Table 1. Position of CPR genes (coding region only) on contigs arranged in their order on chromosomes

Gene	Chromo-some	Sequence Cluster	Contig	Orientation on contig	Exon 1	Exon 2	Exon 3	Exon 4	Exon 5	Ensembl Gene Name
CPR130	X		AAAB01008846	plus	10554469-10554480	10555056-10556108				AGAP000047
CPR129	X		AAAB01008846	plus	9873350-9873358	9874838-9875061	9875143-9875325	9875400-9875715		AGAP000085
CPR128	X		AAAB01008846	minus	8328487-8328579	8328200-8328280	8327990-8328118			AGAP000177
CPR127	X		AAAB01008846	minus	5346310-5346321	5341828-5342083	5341325-5341731			AGAP000344
CPR126	X		AAAB01008846	minus	5324738-5324755	5319804-5319940	5314079-5314358	5312635-5313102	5312336-5312506	error on Ensembl
CPR125	X		AAAB01008963	minus	239247-239258	238701-238957	238204-238618			AGAP000820
CPR1	2R	2RA	AAAB01008987	plus	8582472-8582483	8582579-8583181				AGAP001664
CPR2	2R	2RA	AAAB01008987	plus	8579196-8579207	8579299-8580027				AGAP001665
CPR3	2R	2RA	AAAB01008987	plus	8576336-8576347	8576438-8577163				AGAP001666
CPR4	2R	2RA	AAAB01008987	plus	8572877-8572888	8572979-8573707				AGAP001667
CPR5	2R	2RA	AAAB01008987	plus	8570136-8570147	8570229-8570957				AGAP001668
CPR6	2R	2RA	AAAB01008987	minus	8566158-8566169	8565451-8566080				AGAP001669
CPR7	2R		AAAB01008859	minus	7391947-7392086	7391612-7391867				error on Ensembl
CPR8	2R		AAAB01008859	plus	7389611-7389783	7389911-7390151				AGAP002613
CPR9	2R		AAAB01008859	minus	5251386-5251400	5250889-5251311	5250649-5250810			AGAP002726
CPR10	2R		AAAB01008859	plus	816286-816297	816372-816962				AGAP002994
CPR114	2R		AAAB01008851	minus	663323-663334	662474-662956				AGAP003375
CPR154	2R	2RB	AAAB01008851	plus	no genomic data	no genomic data	<665857-666125			not on Ensembl
CPR115	2R	2RB	AAAB01008851	plus	668683-668694	668798-669058	669138-669425			AGAP003377
CPR116	2R		AAAB01008851	minus	670529-670534	670249-670450	669916-670094			AGAP003378
CPR117	2R	2RB	AAAB01008851	plus	674806-674817	674920-675108	675188-675475			AGAP003379
CPR118	2R	2RB	AAAB01008851	plus	678604-678615	678720-679001	679081-679368			AGAP003380
CPR119	2R	2RB	AAAB01008851	plus	681901-681912	682016-682297	682377-682664			AGAP003381
CPR120	2R	2RB	AAAB01008851	plus	685298-685309	685413-685673	685753-686040			AGAP003382
CPR121	2R	2RB	AAAB01008851	plus	690165-690176	690280-690561	690641-690928			AGAP003383
CPR122	2R	2RB	AAAB01008851	plus	693651-693662	693736-693888	693972-694223			AGAP003384
CPR123	2R	2RB	AAAB01008851	plus	697308-697319	697430-697597	697679-697966			AGAP003385
CPR124	2R		AAAB01008851	minus	733511-733525	732612-733331				AGAP003390
DUPL A	*2R*		*AAAB01008851*	*minus*	*741422-741433*	*740568-741050*				
DUPL B	*2R*		*AAAB01008851*	*plus*	*749094-749105*	*749209-749490*	*749570-749857*			
DUPL C	*2R*		*AAAB01008851*	*plus*	*753088-753099*	*753203-753484*	*753564-753851*			
DUPL D	*2R*		*AAAB01008851*	*plus*	*764935-764946*	*765200-765289*	*765372-765659*			
DUPL E	*2R*		*AAAB01008851*	*plus*	*768455-768466*	*768540-768692*	*768776-769027*			
CPR11	2L		AAAB01008960	plus	1777672-1777686	1778318-1778746				AGAP005451
CPR12	2L		AAAB01008960	minus	1779841-1780281					AGAP005453
CPR13	2L		AAAB01008960	plus	1783590-1784030					AGAP005454
CPR14	2L		AAAB01008960	plus	1796179-1796184	1796343-1796705				AGAP005455

Gene	Chromo-some	Sequence Cluster	Contig	Orientation on contig	Exon 1	Exon 2	Exon 3	Exon 4	Exon 5	Ensembl Gene Name
CPR15	2L		AAAB01008960	plus	1814141-1814152	1814811-1815212				AGAP005456
CPR16	2L		AAAB01008960	plus	1853327-1853338	1856517-1856915				AGAP005459
CPR17	2L	2LA	AAAB01008960	minus	10357172-10357315	10356704-10357093				AGAP005966
CPR18	2L	2LA	AAAB01008960	plus	10358265-10358396	10358485-10358874				AGAP005967
CPR19	2L	2LA	AAAB01008960	minus	10360692-10360817	10360240-10360620				AGAP005968
CPR20	2L	2LA	AAAB01008960	plus	10361565-10361690	10361780-10362079				AGAP005969
CPR138	2L		AAAB01008960	minus	10661715-10661732	10657752-10657872	10656984-10657661	10656984-10657661		AGAP005995
CPR21	2L		AAAB01008960	minus	10666876-10667003	10666201-10666390				AGAP005996
CPR22	2L		AAAB01008960	minus	10671723-10671838	10671424-10671628				AGAP005997
CPR23	2L		AAAB01008960	minus	10677345-10677469	10677030-10677219				AGAP005998
CPR24	2L		AAAB01008960	minus	10679844-10680173					AGAP005999
CPR25	2L		AAAB01008960	minus	10680968-10681282					AGAP006000
CPR26	2L		AAAB01008960	minus	10682440-10682448	10682244-10682368	10681987-10682173			AGAP006001
CPR137	2L		AAAB01008960	plus	10686578-10686720	10686798-10687032				AGAP006002
CPR27	2L		AAAB01008960	minus	10688047-10688055	10687849-10687964	10687528-10687774			AGAP006003
CPR102	2L		AAAB01008960	minus	10689721-10689729	10689528-10689643	10689212-10689461			AGAP006004
CPR103	2L		AAAB01008960	minus	10690842-10690850	10690639-10690766	10690290-10690557			AGAP006005
CPR104	2L		AAAB01008960	minus	10691710-10691721	10691526-10691647	10691187-10691448			AGAP006006
CPR28	2L		AAAB01008960	plus	10692171-10692173	10692263-10692610				AGAP006007
CPR29	2L		AAAB01008960	plus	10693750-10693752	10693845-10694177				AGAP006008
CPR30	2L		AAAB01008960	minus	10696860-10696871	10695822-10695931	10695519-10695702			AGAP006009
CPR105	2L		AAAB01008960	minus	10701141-10701152	10700177-10700482				AGAP006010
CPR31	2L		AAAB01008960	plus	10703957-10703965	10704049-10704155	10704221-10704407			AGAP006011
CPR32	2L		AAAB01008960	minus	10710264-10710272	10709730-10710191				AGAP006012
CPR33	2L		AAAB01008960	plus	10714095-10714103	10714225-10714659				AGAP006013
CPR106	2L		AAAB01008960	minus	12671685-12671699	12671142-12671579				AGAP006095
CPR135	2L		AAAB01008960	minus	14801452-14801676	14801307-14801366	14800893-14801018	14798833-14799192		AGAP006261
CPR139	2L		AAAB01008960	minus	15586045-15586065	15582279-15582447	15581000-15582210			error on Ensembl
CPR70	2L		AAAB01008960	minus	15591086-15591091	15590668-15590808	15590285-15590557			error on Ensembl
CPR71	2L		AAAB01008960	minus	15629835-15629852	15628819-15629406				AGAP006321
CPR144	2L		AAAB01008960	plus	16608902-16609008	16609105-16609245	16610520-16610897	16610992-16612132		AGAP006369
CPR134	2L		AAAB01008960	plus	19107741-19107776	19115443-19115688	19117338-19118153			AGAP006497
CPR72	2L	2LC	AAAB01008960	minus	20656407-20656415	20655935-20656342				AGAP006597
CPR60	2L		AAAB01008807	minus	10313575-10313586	10311605-10311787	10311285-10311512			AGAP006828
CPR59	2L		AAAB01008807	minus	10290013-10290027	10289769-10289849	10288142-10288684			AGAP006829
CPR58	2L		AAAB01008807	minus	10276251-10276343	10275508-10275604	10273598-10273813	10270788-10270891		AGAP006830
CPR57	2L		AAAB01008807	minus	10252742-10253167					AGAP006831
CPR56	2L		AAAB01008807	minus	10248321-10248326	10247744-10248244				AGAP006833

Gene	Chromo-some	Sequence Cluster	Contig	Orientation on contig	Exon 1	Exon 2	Exon 3	Exon 4	Exon 5	Ensembl Gene Name
CPR69	2L		AAAB01008807	plus	10246487-10246495	10246570-10247304				AGAP006834
CPR101	2L		AAAB01008807	plus	10224952-10224960	10225050-10225499				AGAP006836
CPR55	2L		AAAB01008807	minus	10212524-10212776	10212248-10212453				AGAP006837
CPR68	2L		AAAB01008807	minus	10202835-10202948	10201775-10201967	10201590-10201699			AGAP006838
CPR67	2L		AAAB01008807	minus	10193460-10193468	10192682-10193174	10192354-10192586			AGAP006839
CPR136	2L	2LB	AAAB01008807	minus	10189759-10189767	10189263-10189685				AGAP006840
CPR54	2L	2LB	AAAB01008807	minus	10187757-10187765	10187269-10187691				AGAP006841
CPR53	2L	2LB	AAAB01008807	minus	10185588-10185596	10185100-10185522				AGAP006842
CPR52	2L	2LB	AAAB01008807	minus	10183274-10183282	10182834-10183214				AGAP006843
CPR51	2L	2LB	AAAB01008807	plus	10180688-10180696	10180762-10181184				AGAP006844
CPR50	2L	2LB	AAAB01008807	plus	10178704-10178712	10178778-10179188				AGAP006845
CPR49	2L	2LB	AAAB01008807	minus	10178000-10178008	10177512-10177934				AGAP006846
CPR48	2L	2LB	AAAB01008807	plus	10176039-10176047	10176123-10176503				AGAP006847
CPR47	2L	2LB	AAAB01008807	minus	10174731-10174739	10174283-10174630				AGAP006848
CPR46	2L	2LC	AAAB01008807	minus	10171772-10172161					AGAP006849
CPR45	2L	2LC	AAAB01008807	minus	10169828-10170217					AGAP006850
CPR44	2L	2LC	AAAB01008807	minus	10168009-10168398					AGAP006851
CPR43	2L	2LC	AAAB01008807	minus	10165733-10166122					AGAP006852
CPR42	2L	2LC	AAAB01008807	plus	10163032-10163403					AGAP006853
CPR41	2L	2LC	AAAB01008807	plus	10161216-10161605					AGAP006854
CPR40	2L	2LC	AAAB01008807	plus	10159272-10159661					AGAP006855
CPR39	2L	2LC	AAAB01008807	plus	10156858-10157244					AGAP006856
CPR38	2L	2LC	AAAB01008807	plus	10154660-10155037					AGAP006857
CPR37	2L	2LC	AAAB01008807	plus	10152542-10152553	10152620-10152997				AGAP006858
CPR66	2L	2LC	AAAB01008807	minus	10151452-10151460	10151014-10151373				AGAP006859
CPR145	2L	2LC	AAAB01008807	plus	10149348-10149356	10149438-10149791				AGAP006860
CPR36	2L	2LC	AAAB01008807	minus	10148135-10148524					AGAP006861
CPR35	2L	2LC	AAAB01008807	minus	10146257-10146625					AGAP006862
CPR65	2L	2LC	AAAB01008807	plus	10144130-10144138	10144209-10144562				AGAP006863
CPR34	2L	2LC	AAAB01008807	minus	10142677-10142685	10142210-10142614				AGAP006864
CPR64	2L		AAAB01008807	plus	10140893-10140901	10140998-10141570				AGAP006865
CPR63	2L		AAAB01008807	minus	10137300-10137314	10136675-10137208				AGAP006866
CPR141	2L		AAAB01008807	minus	10133833-10133847	10127941-10128027	10124662-10125738			AGAP006867
CPR140	2L		AAAB01008807	minus	10094959-10094964	10089252-10091564	10088920-10089171			AGAP006868
CPR111	2L		AAAB01008807	minus	9252638-9253615					AGAP006931
CPR61	2L		AAAB01008807	minus	8023575-8023589	8022975-8023412				AGAP007040
CPR62	2L		AAAB01008807	minus	7977482-7977496	7976961-7977398				AGAP007042
CPR110	3R		AAAB01008984	minus	3268794-3268802	3268266-3268346	3267315-3267431	3266880-3267239		AGAP008960

Gene	Chromo-some	Sequence Cluster	Contig	Orientation on contig	Exon 1	Exon 2	Exon 3	Exon 4	Exon 5	Ensembl Gene Name
CPR73	3R		AAAB01008980	minus	8451598-8451609	8451238-8451380	8450774-8451119			AGAP009868
CPR74	3R		AAAB01008980	minus	8454868-8454998	8454583-8454753	8454424-8454490			AGAP009869
CPR151	3R		AAAB01008980	plus	8458489-8458779	8458854-8459024				not on Ensembl
CPR75	3R		AAAB01008980	minus	8463766-8463774	8462967-8463088	8461956-8462229			AGAP009871
CPR133	3R		AAAB01008980	plus	8476297-8476314	8476437-8476716	8476879-8477576			AGAP009872
CPR153	3R		AAAB01008980	plus	8485119-8485136	8485259-8485547	8485710-8486407			not on Ensembl
CPR76	3R		AAAB01008980	minus	8503613-8503627	8502997-8503550	8502763-8502928	8502595-8502666		AGAP009874
CPR77	3R		AAAB01008980	minus	8506756-8506764	8506435-8506617	8506167-8506355			AGAP009875
CPR78	3R		AAAB01008980	minus	8513681-8513692	8513400-8513606	8510370-8510564			AGAP009876
CPR79	3R		AAAB01008980	minus	8524541-8524549	8519751-8520051	8519442-8519691	8518777-8519377		AGAP009877
CPR80	3R		AAAB01008980	minus	8540505-8540525	8540142-8540441	8539106-8539346	8538801-8538991		AGAP009878
CPR81	3R		AAAB01008980	plus	8577320-8577328	8578875-8579261				AGAP009879
CPR82	3R	3RA	AAAB01008980	plus	12926957-12926968	12927071-12927802				AGAP010095
CPR107	3R	3RA	AAAB01008980	minus	12982135-12982146	12981541-12982071				AGAP010097
CPR83	3R	3RA	AAAB01008980	minus	12987382-12987393	12986780-12987247				AGAP010098
CPR108	3R	3RA	AAAB01008980	minus	12991560-12991571	12991102-12991497				AGAP010099
CPR84	3R	3RA	AAAB01008980	plus	12992516-12992527	12992600-12992995				AGAP010100
CPR85	3R		AAAB01008980	minus	12997665-12997676	12997076-12997606				AGAP010101
CPR155	3R		AAAB01008980	plus	12998352-12998807					not on Ensembl
CPR156	3R	3RB	AAAB01008980	plus	13000537-13000548	13000624-13000989				
CPR148	3R	3RB	AAAB01008980	plus	13001846-13001857	13001921-13002307				AGAP010102
CPR86	3R	3RB	AAAB01008980	plus	13005010-13005021	13005085-13005471				AGAP010103
CPR87	3R	3RB	AAAB01008980	plus	13010165-13010176	13010240-13010626				AGAP010104
CPR88	3R	3RB	AAAB01008980	plus	13012775-13012783	13012851-13013216				AGAP010105
CPR89	3R	3RB	AAAB01008980	plus	13015454-13015465	13015529-13015915				AGAP010106
CPR90	3R	3RB	AAAB01008980	plus	13019400-13019411	13019475-13019861				AGAP010107
CPR91	3R	3RB	AAAB01008980	plus	13024421-13024432	13024496-13024858				AGAP010108
CPR150	3R		AAAB01008980	plus	no genomic data	<13037505-13037944				not on Ensembl
CPR92	3R	3RC	AAAB01008980	minus	13045870-13045881	13045116-13045781				AGAP010112
CPR93	3R	3RC	AAAB01008980	minus	13048728-13048739	13047956-13048639				AGAP010113
CPR94	3R	3RC	AAAB01008980	minus	13051988-13051999	13051215-13051898				AGAP010114
CPR109	3R	3RC	AAAB01008980	plus	13065776-13065787	13065876-13066559				AGAP010116
CPR95	3R	3RC	AAAB01008980	plus	13070614-13070625	13070714-13071409				AGAP010117
Pseudo3Ra	*3R*		*AAAB01008980*	*minus*	*13074670-130*	*13074812-13075360*				
CPR96	3R	3RC	AAAB01008980	plus	13075392-13075403	13075492-13076163				AGAP010119
CPR97	3R	3RC	AAAB01008980	plus	13082869-13082880	13082969-13083676				AGAP010120
CPR149	3R	3RB	AAAB01008980	plus	13084944-13084955	13085019-13085381				AGAP010121
CPR132	3R		AAAB01008980	plus	13092305-13092316	13092381-13093442				AGAP010122

Gene	Chromo-some	Sequence Cluster	Contig	Orientation on contig	Exon 1	Exon 2	Exon 3	Exon 4	Exon 5	Ensembl Gene Name
CPR131	3R		AAAB01008980	plus	13094742-13094753	13094832-13095383				AGAP010123
CPR98	3R		AAAB01008980	plus	13097846-13097857	13097966-13098535				AGAP010124
CPR142	3R	3RC	AAAB01008980	minus	13107454-13107465	13106670-13107365				AGAP010126
CPR99	3R	3RC	AAAB01008980	minus	13110315-13110326	13109543-13110226				AGAP010127
CPR100	3R	3RC	AAAB01008980	plus	13114949-13114960	13115049-13115732				AGAP010128
CPR112	3L		AAAB01008849	plus	2175870-2175931	2175995-2176427				AGAP010369
CPR143	3L		AAAB01008848	plus	1203199-1203204	1202832-1203037	1202453-1202672	1201187-1202233		error on Ensembl
CPR113	3L		AAAB01008823	plus	2088930-2088944	2088449-2088846	2087057-2087269	2086628-2086970		AGAP010887
Pseudo3La	*3L*		*AAAB01008823*	*minus*	*2734925-2734933*	*2734387-2734489*	*2734102-2734319*			
CPR147	UNKN		AAAB01008831	plus	179292-179297	182205-182612	183107-183238			AGAP012462
CPR146	UNKN		AAAB01008959	minus	136928-136939	113042-113221	112783-112974	112641-112718		AGAP012466
CPR152	UNKN		AAAB01008818	minus	64935-64970	63083-63364	62382-63017			not on Ensembl

Information about genes that were not used is in italics. Tandem arrays are shown in alternating shades of gray. Sequence clusters are shown in color. Genes lacking genomic data were based in part on RACE products with number of exons estimated.
