# Supplementary material for: Annotation and analysis of a large cuticular protein family with the R&R Consensus in Anopheles gambiae
Source: BMC Genomics. 2008 Jan 18;9:22. doi: 10.1186/1471-2164-9-22 (PMC2259329; doi:10.1186/1471-2164-9-22)
Supplement: Additional file 2 — Supplementary Table 2. General information about CPR genes. [file 1471-2164-9-22-S2.PDF]

Supplementary Table 2. General information about CPR genes.

Name	Chrom	Ch. orient.	RR Class	# exons	exon 1	intron 1/2	exon 2	intron 2/3	exon 3	intron 3/4	exon 4	intron 4/5	exon 5
CPR130	X	minus	RR-3?	2	12	575	1053						
CPR129	X	minus	RR-1	4	9	1479	224	81	183	74	316		
CPR128	X	plus	RR-1?	3	93	206	81	81	129				
CPR127	X	plus	RR-1?	3	12	4226	256	96	407				
CPR126	X	plus	RR-1	5	18	4797	137	~5445	280	976	468	128	171
CPR125	X	plus	RR-1	3	12	289	257	82	415				
CPR1	2R	minus	RR-2	2	12	95	603						
CPR2	2R	minus	RR-2	2	12	91	729						
CPR3	2R	minus	RR-2	2	12	90	726						
CPR4	2R	minus	RR-2	2	12	90	729						
CPR5	2R	minus	RR-2	2	12	81	729						
CPR6	2R	plus	RR-2	2	12	77	630						
CPR7	2R	plus	RR-1	2	140	79	256						
CPR8	2R	minus	RR-1	2	173	127	241						
CPR9	2R	plus	RR-1	3	15	74	423	78	162				
CPR10	2R	minus	RR-2	2	12	74	591						
CPR114	2R	minus	RR-2	2	12	366	483						
CPR154	2R	plus	RR-2	3 est.	no data	no data	189 est	no data	288				
CPR115	2R	plus	RR-2	3	12	103	261	79	288				
CPR116	2R	minus	RR-2	3	6	78	202	154	179				
CPR117	2R	plus	RR-2	3	12	102	189	79	288				
CPR118	2R	plus	RR-2	3	12	104	282	79	288				
CPR119	2R	plus	RR-2	3	12	103	282	79	288				
CPR120	2R	plus	RR-2	3	12	103	261	79	288				
CPR121	2R	plus	RR-2	3	12	103	282	79	288				
CPR122	2R	plus	RR-2	3	12	73	153	83	252				
CPR123	2R	plus	RR-2	3	12	110	168	81	288				
CPR124	2R	minus	RR-2	2	15	179	720						
CPR11	2L	plus	RR-1	2	15	631	429						
CPR12	2L	minus	RR-1	1	441								
CPR13	2L	plus	RR-1	1	441								
CPR14	2L	plus	RR-1	2	6	158	363						

Name	Chrom	Ch. orient.	RR Class	# exons	exon 1	intron 1/2	exon 2	intron 2/3	exon 3	intron 3/4	exon 4	intron 4/5	exon 5
CPR15	2L	plus	RR-1	2	12	658	402						
CPR16	2L	plus	RR-1	2	12	3178	399						
CPR17	2L	minus	RR-2	2	144	78	390						
CPR18	2L	plus	RR-2	2	132	88	390						
CPR19	2L	minus	RR-2	2	126	71	381						
CPR20	2L	plus	RR-2	2	126	89	300						
CPR138	2L	minus	RR-1?	4	18	3842	121	90	678	153	395		
CPR21	2L	minus	RR-1	2	128	485	190						
CPR22	2L	minus	RR-1	2	116	94	205						
CPR23	2L	minus	RR-1	2	125	125	190						
CPR24	2L	minus	RR-1	1	330								
CPR25	2L	minus	RR-1	1	315								
CPR26	2L	minus	RR-1	3	9	71	125	70	187				
CPR137	2L	plus	RR-1	2	143	77	235						
CPR27	2L	minus	RR-1	3	9	82	116	74	247				
CPR102	2L	minus	RR-1	3	9	77	116	66	250				
CPR103	2L	minus	RR-1	3	9	75	128	81	268				
CPR104	2L	minus	RR-1	3	12	62	122	77	262				
CPR28	2L	plus	RR-1	2	3	89	348						
CPR29	2L	plus	RR-1	2	3	92	333						
CPR30	2L	minus	RR-1	3	12	928	110	119	184				
CPR105	2L	minus	RR-1	2	12	658	306						
CPR31	2L	plus	RR-1	3	9	83	107	65	187				
CPR32	2L	minus	RR-1	2	9	72	462						
CPR33	2L	plus	RR-1	2	9	121	435						
CPR106	2L	minus	RR-1	2	15	105	438						
CPR135	2L	minus	RR-2	4	225	85	60	288	126	1700	360		
CPR139	2L	minus	RR-1?	3	21	3597	169	68	1211				
CPR70	2L	minus	RR-2	3	6	277	141	110	273				
CPR71	2L	minus	RR-2	2	18	428	588						
CPR144	2L	plus	3 regions	4	107	96	141	1274	378	94	1141		
CPR134	2L	plus	RR-1?	3	36	7666	246	1649	816				
CPR72	2L	minus	RR-2	2	9	64	408						

Name	Chrom	Ch. orient.	RR Class	# exons	exon 1	intron 1/2	exon 2	intron 2/3	exon 3	intron 3/4	exon 4	intron 4/5	exon 5
CPR60	2L	plus	RR-2	3	12	1787	183	92	228				
CPR59	2L	plus	RR-2	3	15	163	81	1084	543				
CPR58	2L	plus	RR-2	4	93	646	97	1694	216	2706	104		
CPR57	2L	plus	RR-2	1	426								
CPR56	2L	plus	RR-2	2	6	76	501						
CPR69	2L	minus	RR-2	2	9	74	735						
CPR101	2L	minus	RR-2	2	9	89	450						
CPR55	2L	plus	RR-2	2	253	70	206						
CPR68	2L	plus	RR-2	3	114	867	193	75	110				
CPR67	2L	plus	RR-2	3	9	285	493	95	233				
CPR136	2L	plus	RR-2	2	9	73	423						
CPR54	2L	plus	RR-2	2	9	65	423						
CPR53	2L	plus	RR-2	2	9	65	423						
CPR52	2L	plus	RR-2	2	9	59	381						
CPR51	2L	minus	RR-2	2	9	65	423						
CPR50	2L	minus	RR-2	2	9	65	411						
CPR49	2L	plus	RR-2	2	9	65	423						
CPR48	2L	minus	RR-2	2	9	75	381						
CPR47	2L	plus	RR-2	2	9	100	348						
CPR46	2L	plus	RR-2	1	390								
CPR45	2L	plus	RR-2	1	390								
CPR44	2L	plus	RR-2	1	390								
CPR43	2L	plus	RR-2	1	390								
CPR42	2L	minus	RR-2	1	372								
CPR41	2L	minus	RR-2	1	390								
CPR40	2L	minus	RR-2	1	390								
CPR39	2L	minus	RR-2	1	387								
CPR38	2L	minus	RR-2	1	378								
CPR37	2L	minus	RR-2	2	12	66	378						
CPR66	2L	plus	RR-2	2	9	78	360						
CPR145	2L	minus	RR-2	2	9	81	354						
CPR36	2L	plus	RR-2	1	390								
CPR35	2L	plus	RR-2	1	369								

Name	Chrom	Ch. orient.	RR Class	# exons	exon 1	intron 1/2	exon 2	intron 2/3	exon 3	intron 3/4	exon 4	intron 4/5	exon 5
CPR65	2L	minus	RR-2	2	9	70	354						
CPR34	2L	plus	RR-2	2	9	62	405						
CPR64	2L	minus	RR-2	2	9	96	573						
CPR63	2L	plus	RR-2	2	15	91	534						
CPR141	2L	plus	RR-2	3	15	5805	87	2202	1077				
CPR140	2L	plus	RR-2	3	6	3394	2313	80	252				
CPR111	2L	plus	RR-3?	1	978								
CPR61	2L	plus	RR-1	2	15	162	438						
CPR62	2L	plus	RR-1	2	15	83	438						
CPR110	3R	minus	RR-2	4	9	447	81	834	117	75	360		
CPR73	3R	minus	RR-1	3	12	217	143	118	346				
CPR74	3R	minus	RR-1	3	131	114	171	92	67				
CPR151	3R	plus	RR-1	2	291	74	171						
CPR75	3R	minus	RR-1	3	9	677	122	737	274				
CPR133	3R	plus	RR-1	3	18	122	280	162	698				
CPR153	3R	plus	RR-1	3	18	122	289	162	698				
CPR76	3R	minus	RR-1	4	15	62	554	68	166	96	72		
CPR77	3R	minus	RR-1	3	9	138	183	79	189				
CPR78	3R	minus	RR-1	3	12	74	207	2835	195				
CPR79	3R	minus	RR-1	4	9	4489	301	59	250	64	601		
CPR80	3R	minus	RR-1	4	21	63	300	795	241	114	191		
CPR81	3R	plus	RR-1	2	9	1546	387						
CPR82	3R	plus	RR-2	2	12	102	732						
CPR107	3R	minus	RR-2	2	12	63	531						
CPR83	3R	minus	RR-2	2	12	134	468						
CPR108	3R	minus	RR-2	2	12	62	396						
CPR84	3R	plus	RR-2	2	12	72	396						
CPR85	3R	minus	RR-2	2	12	58	531						
CPR155	3R	plus	RR-2	1	456								
CPR156	3R	plus	RR-2	2	12	75	366						
CPR148	3R	plus	RR-2	2	12	63	387						
CPR86	3R	plus	RR-2	2	12	63	387						
CPR87	3R	plus	RR-2	2	12	63	387						

Name	Chrom	Ch. orient.	RR Class	# exons	exon 1	intron 1/2	exon 2	intron 2/3	exon 3	intron 3/4	exon 4	intron 4/5	exon 5
CPR88	3R	plus	RR-2	2	9	67	366						
CPR89	3R	plus	RR-2	2	12	63	387						
CPR90	3R	plus	RR-2	2	12	63	387						
CPR91	3R	plus	RR-2	2	12	63	363						
CPR150	3R	plus	RR-2	2est	no data	no data	>440						
CPR92	3R	minus	RR-2	2	12	88	666						
CPR93	3R	minus	RR-2	2	12	88	684						
CPR94	3R	minus	RR-2	2	12	89	684						
CPR109	3R	plus	RR-2	2	12	88	684						
CPR95	3R	plus	RR-2	2	12	88	696						
CPR96	3R	plus	RR-2	2	12	88	672						
CPR97	3R	plus	RR-2	2	12	88	708						
CPR149	3R	plus	RR-2	2	12	63	363						
CPR132	3R	plus	RR-2	2	12	64	1062						
CPR131	3R	plus	RR-2	2	12	78	552						
CPR98	3R	plus	RR-2	2	12	108	570						
CPR142	3R	minus	RR-2	2	12	88	696						
CPR99	3R	minus	RR-2	2	12	88	684						
CPR100	3R	plus	RR-2	2	12	88	684						
CPR112	3L	minus	RR-3?	2	62	63	433						
CPR143	3L	minus	RR-2	4	6	161	206	159	220	219	1047		
CPR113	3L	plus	RR-1	4	15	83	398	1179	213	86	343		
CPR147	UNKN	plus	RR-2	3	6	2907	408	494	132				
CPR146	UNKN	minus	RR-2	4	12	~24 kb	180	67	192	64	78		
CPR152	UNKN	minus	RR-2	3	36	1570	282	65	636				

Tandem arrays are showin in alternating shades of gray. Sequence clusters are highlighted in color.
