# Supplementary material for: Annotation and analysis of a large cuticular protein family with the R&R Consensus in Anopheles gambiae
Source: BMC Genomics. 2008 Jan 18;9:22. doi: 10.1186/1471-2164-9-22 (PMC2259329; doi:10.1186/1471-2164-9-22)
Supplement: Additional file 3 — Supplementary Table 3. Evidence that supports annotation. [file 1471-2164-9-22-S3.PDF]

Supplementary Table 3 Evidence that supports annotation.

Name	Chrom	TATA[a]	Space[b]	INR[c]	DPE[d]	polyA[e]	Support[f]	qRT-PCR[g]	Unique Pep[h]	Shared Pep[i]
CPR130	X	yes	26	ACAGT		nnnn	ESTs partial; 5' RACE	+++	29%	
CPR129	X	yes	27	ttagt		yes	RT-PCR	++	4%	
CPR128	X	yes	27	ccagt		yes	EST 100%	++		
CPR127	X	tatata	22	***gtacg***		yes	5' RACE	++	37%	
CPR126	X	yes	26	**TCAGT**		yes	EST partial	++	33%	
CPR125	X	yes	25	TCAGT		no	ESTs 100%	++++	33%	
CPR1	2R	yes	25	ACAGT		no	aatata	++++	39%	48%
CPR2	2R	yes	25	ACAGT		yes	none	++++		90%
CPR3	2R	yes	25	ACAGT		aataca	EST 100%	++++	7%	83%
CPR4	2R	yes	25	ACAGT		aataca	none	++++		90%
CPR5	2R	yes	25	ACAGT		aataca	none	++++		90%
CPR6	2R	yes	25	ACAGT		yes	EST 100%	++++	23%	65%
CPR7	2R	yes	26	TCAGT		yes	RT-PCR	++		
CPR8	2R	yes	24	TCATT		yes	EST 100%	+++	43%	
CPR9	2R	yes	27	ctatg		yes	ESTs partial	++	32%	
CPR10	2R	yes	27	TCATT		yes	EST; RT-PCR	+++	40%	
CPR114	2R	tattttata	23	tcata		nnnn	EST partial	+++	9%	
CPR154	2R	no data		***tcaca***		yes	5' RACE	++++		77%
CPR115	2R	yes	26	***tcaca***		nnnn	5' RACE	++++		54%
CPR116	2R	yes	25	tcaaa		yes	EST 100%	+++	18%	
CPR117	2R	yes	26	tcaca		yes	none	++++		77%
CPR118	2R	yes	26	tcaca		yes	none	++++		69%
CPR119	2R	yes	27	tcaca		yes	none	+++		69%
CPR120	2R	yes	26	***tcaca***		yes	5' RACE	+++	12%	59%
CPR121	2R	yes	25	tcaca		yes	none	++++		69%
CPR122	2R	yes	24	acatt		yes	EST 100%	++++	55%	21%
CPR123	2R	yes	30	tcagc		yes	none	++++		70%
CPR124	2R	yes	27	TCAGT		aataca	ESTs partial	+++	62%	
CPR11	2L	yes	26	TCAGT		yes	RT-PCR	++++	14%	
CPR12	2L	yes	26	TCAGT		yes	RT-PCR	NSP		15%
CPR13	2L	yes	24	TCATT		yes	none	NSP		15%
CPR14	2L	tataat	27	tcaag		aatata	none	++		
CPR15	2L	yes	25	TCAGT		yes	ESTs 100%	+++	17%	5%
CPR16	2L	yes	25	TCAGT		no	ESTs 100%	+++	68%	
CPR17	2L	yes	26	TCAGT		yes	none	++		18%
CPR18	2L	yes	29	TCAGT		aataca	none	+++		18%

Name	Chrom	TATA[a]	Space[b]	INR[c]	DPE[d]	polyA[e]	Support[f]	qRT-PCR[g]	Unique Pep[h]	Shared Pep[i]
CPR19	2L	yes	27	TCAGT		yes	none	++		19%
CPR20	2L	yes	26	tcact		yes	none	+++		16%
CPR138	2L	no		***tgaag***	26-ggatg	aataca	5' RACE	+	5%	
CPR21	2L	yes	26	TCAGT		aataca	ESTs partial	++++	54%	
CPR22	2L	yes	26	caagt		yes	none	+++	13%	
CPR23	2L	yes	26	TCAGT		yes	ESTs 100%	++++	28%	
CPR24	2L	yes	26	TCAGT		yes	EST 100%	+++	14%	
CPR25	2L	yes	26	ttagt		yes	none	+++	13%	
CPR26	2L	yes	27	ttagt		yes	EST 100%	+++	7%	
CPR137	2L	no		***ttatg***	29 ggtgc	yes	5', 3' RACE	+	8%	
CPR27	2L	no		gtacg	31 ggacg	yes	EST 100%	+		6%
CPR102	2L	no		GCAGT	31ggaaa	yes	none	+		6%
CPR103	2L	yes	27	atata		yes	EST partial	+	11%	
CPR104	2L	no		TCAGT	29 ggttg	yes	none	++	6%	
CPR28	2L	tataag	26	gcaac		yes	none	+		
CPR29	2L	yes	24	ttacc		yes	none	++	14%	
CPR30	2L	yes	25	ttatt		yes	EST 100%	+++		
CPR105	2L	yes	24	acatt		yes	none	+++	8%	
CPR31	2L	yes	25	TCAGT		yes	none	+++		
CPR32	2L	yes	26	TCAGT		yes	none	+		6%
CPR33	2L	yes	27	TCAGT		yes	none	++		6%
CPR106	2L	yes	27	ACAGT		yes	ESTs 100%	+++	20%	
CPR135	2L	yes	27	TCATT		yes	ESTs	++	64%	
CPR139	2L	yes	26	***TCAGT***		yes	5' RACE	+	6%	
CPR70	2L	yes	26	TCAGT		no	cDNA 100%	++++	68%	11%
CPR71	2L	yes	26	ACAGT		yes	EST partial	++		
CPR144	2L	yes	25	ACAGT		yes	ESTs; 5' RACE; RT-PCR	+	7%	
CPR134	2L	yes	27	ttatt		yes	5' RACE	+++	31%	
CPR72	2L	yes	26	TCATT		yes	none	+++		18%
CPR60	2L	yes	26	TCAGT		no	EST 100%	++++	15%	20%
CPR59	2L	yes	26	TCATT		no	EST 100%	+++	39%	
CPR58	2L	yes	25	ACAGT		yes	EST 100%	+++	44%	17%
CPR57	2L	yes	24	TCAGT		yes	EST 100%	+	14%	
CPR56	2L	yes	26	TCAGT		yes	ESTs partial	++	8%	
CPR69	2L	yes	26	ccatc		yes	EST 100%	++	3%	3%
CPR101	2L	yes	26	GCAGT		yes	none	+	7%	
CPR55	2L	yes	26	TCAGT		yes	none	++	15%	

Name	Chrom	TATA[a]	Space[b]	INR[c]	DPE[d]	polyA[e]	Support[f]	qRT-PCR[g]	Unique Pep[h]	Shared Pep[i]
CPR68	2L	no		***cgaac***	29 gatcg	yes	EST; 5' RACE	+		
CPR67	2L	yes	26	TCAGT		yes	EST partial	++		
CPR136	2L	yes	27	TCAGT		yes	none	+++		10%
CPR54	2L	yes	26	TCAGT		yes	none	++		10%
CPR53	2L	yes	26	TCAGT		yes	none	++		10%
CPR52	2L	yes	26	TCAGT		aatata	none	++		22%
CPR51	2L	yes	26	TCAGT		yes	none	++		10%
CPR50	2L	yes	26	TCAGT		yes	none	++		11%
CPR49	2L	yes	26	TCAGT		yes	none	++		10%
CPR48	2L	yes	26	TCAGT		yes	none	++		22%
CPR47	2L	yes	27	TCAGT		aataca	EST partial	+++		6%
CPR46	2L	tatttaa	24	ACAGT		yes	none	++		19%
CPR45	2L	tatttaa	25	TCAGT		yes	none	++		19%
CPR44	2L	tatttaa	28	TCAGT		yes	none	+		19%
CPR43	2L	tatttaa	25	TCAGT		yes	none	++		19%
CPR42	2L	tatttaa	25	TCAGT		yes	none	++		20%
CPR41	2L	tatttaa	25	TCAGT		yes	none	++		19%
CPR40	2L	tatttaa	25	ACAGT		yes	EST partial	++		19%
CPR39	2L	tatttaa	25	TCAGT		yes	none	++		19%
CPR38	2L	tatttaa	25	TCAGT		yes	EST 100%	++	11%	7%
CPR37	2L	yes	26	TCAGT		yes	none	+		7%
CPR66	2L	yes	26	TCAGT		yes	none	++		20%
CPR145	2L	tatata	25	TCAGT		yes	none	++		8%
CPR36	2L	tatttaa	25	TCAGT		yes	none	++		19%
CPR35	2L	tatttaa	24	TCAGT		yes	none	++		20%
CPR65	2L	tatata	25	TCATT		yes	none	NSP		20%
CPR34	2L	yes	26	TCATT		yes	EST 100%	++		18%
CPR64	2L	yes	25	ccatt		no	EST 100%	+		
CPR63	2L	tatata	27	TCAGT		aataca	EST partial	+++		5%
CPR141	2L	yes	24	tgact		yes	ESTs partial	+++	6%	
CPR140	2L	yes	26	TCAGT		no[e]	EST partial; 3' RACE	+++	62%	
CPR111	2L	yes	27	tcaat		yes	EST partial	++++	34%	
CPR61	2L	yes	25	TCAGT		yes	none	+++	15%	
CPR62	2L	yes	27	ccagt		yes	EST 100%	++	20%	
CPR110	3R	no		GCAGT	27 ggttt	yes	none	+++	44%	
CPR73	3R	yes	25	GCAGT		yes	EST 100%	++	9%	

Name	Chrom	TATA[a]	Space[b]	INR[c]	DPE[d]	polyA[e]	Support[f]	qRT-PCR[g]	Unique Pep[h]	Shared Pep[i]
CPR74	3R	yes	26	TCAGT		no	EST 100%	+++	28%	
CPR151	3R	yes	26	GCAGT		yes	none	+++	7%	
CPR75	3R	yes	25	TCATT		yes	EST 100%	++++		
CPR133	3R	yes	26	TCATT		yes	none	NSP		21%
CPR153	3R	yes	26	acatt		yes	none	NSP		21%
CPR76	3R	yes	29	acatt		yes	EST 100%	+++	40%	
CPR77	3R	yes	26	ccaga		yes	EST 100%	+++	6%	
CPR78	3R	yes	27	gcaga		yes	EST partial	++	22%	
CPR79	3R	yes	24	acatt		yes	RT-PCR	++	8%	
CPR80	3R	yes	26	atagt		no	ESTs partial	++	8%	
CPR81	3R	yes	21	TCAGT		no	EST 100%	+++	10%	
CPR82	3R	yes	24	ACAGT		yes	EST partial	+++	24%	13%
CPR107	3R	yes	26	TCAGT		yes	none	++++	15%	32%
CPR83	3R	yes	26	TCAGT		yes	EST 100%	+++	35%	36%
CPR108	3R	yes	26	tcaaa		yes	EST 100%	+++		55%
CPR84	3R	yes	26	ccagt		yes	EST 100%	+++		55%
CPR85	3R	yes	25	ccatt		yes	none	++	11%	
CPR155	3R	yes	25	***TCATT***		aataca	5' RACE	-	11%	
CPR156	3R	yes	26	TCAGT		yes	none	+++		14%
CPR148	3R	yes	25	TCAGT		aatata	none	+++		32%
CPR86	3R	yes	24	TCAGT		yes	none	+++		32%
CPR87	3R	yes	24	TCAGT		aatata	none	+		32%
CPR88	3R	yes	36	GCAGT		yes	none	+++		34%
CPR89	3R	yes	24	TCAGT		yes	none	+++		32%
CPR90	3R	yes	24	TCAGT		yes	none	+++		32%
CPR91	3R	yes	26	TCAGT		yes	none	NSP		35%
CPR150	3R	no data		***TCATT***		yes	5' RACE	+++		39%
CPR92	3R	yes	25	ACAGT		yes	none	+++		19%
CPR93	3R	yes	26	ACAGT		yes	Dotson cDNA 5d	+++		27%
CPR94	3R	yes	26	ACAGT		yes	none	NSP		27%
CPR109	3R	yes	26	ACAGT		yes	none	+++		27%
CPR95	3R	yes	26	ACAGT		yes	none	+++		27%
CPR96	3R	yes	26	ACAGT		yes	~AGCP2a mRNA	++		25%
CPR97	3R	yes	26	ACAGT		yes	AGCP2b mRNA	+++		26%
CPR149	3R	no data		no data		yes	none	+++		35%
CPR132	3R	yes	26	TCAGT		yes	EST	+++	12%	
CPR131	3R	yes	26	TCAGT		yes	EST	+++	59%	

Name	Chrom	TATA[a]	Space[b]	INR[c]	DPE[d]	polyA[e]	Support[f]	qRT-PCR[g]	Unique Pep[h]	Shared Pep[i]
CPR98	3R	yes	25	TCATT		yes	EST	+++	16%	39%
CPR142	3R	yes	26	ACAGT		yes		NSP		27%
CPR99	3R	yes	26	ACAGT		yes	Dotson cDNA 18a 13a	NSP		27%
CPR100	3R	yes	26	ACAGT		yes	AGCP2c mRNA	+++		27%
CPR112	3L	yes	26	TCAGT		yes	none	-	4%	
CPR143	3L	no	26	*tcacc*	28 gaacc	yes	RT-PCR	++	2%	
CPR113	3L	yes	29	TCAGT		yes	ESTs	++	17%	
CPR147	UNKN	tatata	24	ccata		yes	EST	++	6%	
CPR146	UNKN	yes	29	GCAGT		yes	ESTs	++	45%	
CPR152	UNKN	yes	26	***TCAGT***		yes	5', 3' RACE	++	3%	

[a]Presence of standard TATA box, TATAAA or variant.

[b]Number of nucleotides between end of TATA box and start of INR.

[c]Sequence of INR; bold italics indicate that sequence was confirmed by 5' RACE; capital letters are standard elements [24].

[d]DPE estimated by position downstream of INR, nucleotides include A of INR and start of putative DPE [25].

[e]PolyA addition site within first 500 nucleotides downstream of stop codon, AATAAA, AATACA or AATATA. In addition, 3' RACE revealed that two AATATA sites were used in *CPR140*; they began at 547 and 797 nucleotides after the stop codon.

[f]Support indicates whether there was an EST on the ENSEMBL Web Site or if our laboratory obtained a RT-PCR or RACE product.

[g]qRT-PCR indicates that we obtained a product with the G3 strain of *An. gambiae* [13]. The number of pluses indicates highest $R_0 \times 10^{-7}$ level obtained at any of the stages examined. Log values were used: + (10-99), ++ (100-999), etc. NSP indicates that no specific primers could be designed.

[h]Percent coverage of mature protein by unique peptides from a proteomics analysis of cuticles from the G3 strain.

[i]Percent coverage by peptides shared with other CPR proteins. Proteomics data come from [12 and He, unpublished observations].
