# Supplementary material for: Annotation and analysis of a large cuticular protein family with the R&R Consensus in Anopheles gambiae
Source: BMC Genomics. 2008 Jan 18;9:22. doi: 10.1186/1471-2164-9-22 (PMC2259329; doi:10.1186/1471-2164-9-22)
Supplement: Additional file 4 — Supplementary Table 4. Selected features of CPR proteins. [file 1471-2164-9-22-S4.PDF]

Supplementary Table 4. Selected features of CPR proteins.

Name	Chrom.	Class	Mature AA	Signal	Start of triad	% to start	#C	#M	# H	%H	# K	%K	%H+%K	# G	%G	# A	%A	%G+%A	#P	%P	#Q	%Q	#AAP(AVL)
CPR130	X	RR-3 ?	334	20	20	6.0			38	11.4	14	4.2	15.6	26	7.8	76	22.8	30.5	29	8.7	12	3.6	1
CPR129	X	RR-1	223	20	diad 55	24.7		3	2	0.9	15	6.7	7.6	13	5.8	11	4.9	10.8	22	9.9	9	4.0	
CPR128	X	RR-1 ?	78	22	10	12.8		1			8	10.3	10.3	7	9.0	7	9.0	17.9	5	6.4	4	5.1	
CPR127	X	RR-1 ?	208	16	23	11.1			6	2.9	8	3.8	6.7	15	7.2	15	7.2	14.4	26	12.5	23	11.1	
CPR126	X	RR-1	338	19	40	11.8			12	3.6	17	5.0	8.6	23	6.8	36	10.7	17.5	54	16.0	43	12.7	4
CPR125	X	RR-1	211	16	monad 24	11.4			6	2.8	6	2.8	5.7	17	8.1	23	10.9	19.0	33	15.6	25	11.8	2
CPR1	2R	RR-2	187	17	56	29.9			5	2.7	9	4.8	7.5	7	3.7	49	26.2	29.9	17	9.1	5	2.7	8
CPR2	2R	RR-2	229	17	49	21.4			3	1.3	12	5.2	6.6	7	3.1	55	24.0	27.1	22	9.6	10	4.4	9
CPR3	2R	RR-2	228	17	49	21.5			4	1.8	12	5.3	7.0	7	3.1	55	24.1	27.2	22	9.6	9	3.9	9
CPR4	2R	RR-2	229	17	49	21.4			3	1.3	12	5.2	6.6	7	3.1	55	24.0	27.1	22	9.6	10	4.4	9
CPR5	2R	RR-2	229	17	49	21.4			4	1.7	12	5.2	7.0	7	3.1	55	24.0	27.1	22	9.6	10	4.4	9
CPR6	2R	RR-2	196	17	50	25.5			3	1.5	10	5.1	6.6	7	3.6	49	25.0	28.6	18	9.2	8	4.1	8
CPR7	2R	RR-1	111	20	24	21.6		1	1	0.9	5	4.5	5.4	13	11.7	6	5.4	17.1	3	2.7	5	4.5	
CPR8	2R	RR-1	115	22	33	28.7			1	0.9	5	4.3	5.2	17	14.8	11	9.6	24.3	8	7.0	9	7.8	
CPR9	2R	RR-1	182	17	33	18.1			10	5.5	10	5.5	11.0	9	4.9	23	12.6	17.6	13	7.1	9	4.9	
CPR10	2R	RR-2	179	21	70	39.1			5	2.8	1	0.6	3.4	12	6.7	8	4.5	11.2	5	2.8	27	15.1	
CPR114	2R	RR-2	147	17	59	40.1			13	8.8	8	5.4	14.3	10	6.8	25	17.0	23.8	14	9.5	1	0.7	2
CPR154	2R	RR-2	145	17	43	29.7			3	2.1	7	4.8	6.9	11	7.6	29	20.0	27.6	16	11.0	3	2.1	2
CPR115	2R	RR-2	169	17	67	39.6			6	3.6	7	4.1	7.7	13	7.7	39	23.1	30.8	19	11.2	3	1.8	2
CPR116	2R	RR-2	107	21	21	19.6					2	1.9	1.9	8	7.5	15	14.0	21.5	12	11.2	5	4.7	
CPR117	2R	RR-2	145	17	43	29.7			3	2.1	7	4.8	6.9	11	7.6	29	20.0	27.6	16	11.0	3	2.1	2
CPR118	2R	RR-2	176	17	74	42.0			7	4.0	7	4.0	8.0	14	8.0	41	23.3	31.3	20	11.4	3	1.7	2
CPR119	2R	RR-2	176	17	74	42.0			7	4.0	7	4.0	8.0	14	8.0	41	23.3	31.3	20	11.4	3	1.7	2
CPR120	2R	RR-2	169	17	67	39.6			6	3.6	7	4.1	7.7	13	7.7	39	23.1	30.8	19	11.2	3	1.8	2
CPR121	2R	RR-2	176	17	74	42.0			7	4.0	7	4.0	8.0	14	8.0	41	23.3	31.3	20	11.4	3	1.7	2
CPR122	2R	RR-2	121	17	31	25.6			1	0.8	5	4.1	5.0	10	8.3	23	19.0	27.3	12	9.9	4	3.3	3
CPR123	2R	RR-2	138	17	36	26.1			2	1.4	7	5.1	6.5	11	8.0	26	18.8	26.8	15	10.9	3	2.2	2
CPR124	2R	RR-2	226	18	138	61.1					6	2.7	2.7	5	2.2	73	32.3	34.5	26	11.5	7	3.1	10
CPR11	2L	RR-1	130	17	34	26.2			2	1.5	1	0.8	2.3	14	10.8	18	13.8	24.6	10	7.7	7	5.4	
CPR12	2L	RR-1	129	17	32	24.8		2	8	6.2	4	3.1	9.3	10	7.8	14	10.9	18.6	11	8.5	8	6.2	
CPR13	2L	RR-1	129	17	32	24.8			8	6.2	4	3.1	9.3	10	7.8	14	10.9	18.6	11	8.5	8	6.2	
CPR14	2L	RR-1	106	16	24	22.6			3	2.8	3	2.8	5.7	11	10.4	13	12.3	22.6	9	8.5	7	6.6	
CPR15	2L	RR-1	121	16	23	19.0		1	2	1.7	3	2.5	4.1	12	9.9	14	11.6	21.5	9	7.4	12	9.9	
CPR16	2L	RR-1	120	16	22	18.3			2	1.7	2	1.7	3.3	10	8.3	12	10.0	18.3	10	8.3	12	10.0	
CPR17	2L	RR-2	159	18	60	37.7			7	4.4	15	9.4	13.8	44	27.7	3	1.9	29.6	3	1.9	3	1.9	
CPR18	2L	RR-2	156	17	57	36.5			7	4.5	15	9.6	14.1	42	26.9	3	1.9	28.8	3	1.9	5	3.2	
CPR19	2L	RR-2	151	17	52	34.4			7	4.6	15	9.9	14.6	39	25.8	3	2.0	27.8	3	2.0	4	2.6	
CPR20	2L	RR-2	124	17	55	44.4		1	6	4.8	12	9.7	14.5	31	25.0	3	2.4	27.4	3	2.4	5	4.0	
CPR138	2L	RR-1 ?	385	18	303	78.7	2	5	10	2.6	32	8.3	10.9	40	10.4	20	5.2	15.6	47	12.2	15	3.9	
CPR21	2L	RR-1	89	16	24	27.0		1	2	2.2	5	5.6	7.9	9	10.1	7	7.9	18.0	3	3.4	6	6.7	
CPR22	2L	RR-1	90	16	20	22.2			2	2.2	6	6.7	8.9	8	8.9	6	6.7	15.6	3	3.3	7	7.8	

Name	Chrom.	Class	Mature AA	Signal	Start of triad	% to start	#C	#M	# H	%H	# K	%K	%H+%K	# G	%G	# A	%A	%G+%A	#P	%P	#Q	%Q	#AAP(AVL)
CPR23	2L	RR-1	88	16	23	26.1			1	1.1	2	2.3	3.4	7	8.0	9	10.2	18.2	5	5.7	4	4.5	
CPR24	2L	RR-1	90	19	26	28.9			2	2.2	4	4.4	6.7	8	8.9	8	8.9	17.8	5	5.6	9	10.0	
CPR25	2L	RR-1	87	17	23	26.4			1	1.1	3	3.4	4.6	9	10.3	10	11.5	21.8	4	4.6	5	5.7	
CPR26	2L	RR-1	88	18	24	27.3			1	1.1	1	1.1	2.3	9	10.2	8	9.1	19.3	5	5.7	1	1.1	
CPR137	2L	RR-1	106	19	26	24.5		10	1	0.9	6	5.7	6.6	10	9.4	6	5.7	15.1	2	1.9	5	4.7	
CPR27	2L	RR-1	103	20	19	18.4		1	1	1.0	1	1.0	1.9	11	10.7	4	3.9	14.6	6	5.8	3	2.9	
CPR102	2L	RR-1	104	20	19	18.3		1	1	1.0	2	1.9	2.9	10	9.6	6	5.8	15.4	7	6.7	4	3.8	
CPR103	2L	RR-1	113	21	diad 24	21.2		2	3	2.7	5	4.4	7.1	10	8.8	4	3.5	12.4	7	6.2	3	2.7	
CPR104	2L	RR-1	111	20	diad 24	21.6		1	2	1.8	4	3.6	5.4	12	10.8	7	6.3	17.1	8	7.2	6	5.4	
CPR28	2L	RR-1	98	18	22	22.4					4	4.1	4.1	13	13.3	3	3.1	16.3	7	7.1	3	3.1	
CPR29	2L	RR-1	95	16	22	23.2					3	3.2	3.2	10	10.5	3	3.2	13.7	6	6.3	4	4.2	
CPR30	2L	RR-1	82	19	19	23.2					5	6.1	6.1	7	8.5	3	3.7	12.2	3	3.7	6	7.3	
CPR105	2L	RR-1	89	16	23	25.8			2	2.2	5	5.6	7.9	6	6.7	7	7.9	14.6	4	4.5	3	3.4	
CPR31	2L	RR-1	82	18	18	22.0		3			6	7.3	7.3	5	6.1	6	7.3	13.4	2	2.4	4	4.9	
CPR32	2L	RR-1	136	20	32	23.5			11	8.1	6	4.4	12.5	10	7.4	15	11.0	18.4	13	9.6	11	8.1	
CPR33	2L	RR-1	131	16	28	21.4			15	11.5	7	5.3	16.8	13	9.9	13	9.9	19.8	14	10.7	9	6.9	
CPR106	2L	RR-1	132	18	34	25.8			2	1.5	3	2.3	3.8	11	8.3	16	12.1	20.5	14	10.6	7	5.3	
CPR135	2L	RR-2	239	17	125	52.3			3	1.3	9	3.8	5.0	12	5.0	20	8.4	13.4	25	10.5	59	24.7	
CPR139	2L	RR-1 ?	446	20	48	10.8			8	1.8	11	2.5	4.3	44	9.9	31	7.0	16.8	44	9.9	56	12.6	
CPR70	2L	RR-2	123	16	38	30.9		9	11	8.9	7	5.7	14.6	7	5.7	17	13.8	19.5	12	9.8	3	2.4	1
CPR71	2L	RR-2	182	19	53	29.1			26	14.3	12	6.6	20.9	20	11.0	14	7.7	18.7	6	3.3	14	7.7	
CPR144	2L	3 regions	569	19					13	2.3	23	4.0	6.3	76	13.4	37	6.5	19.9	59	10.4	23	4.0	
CPR134	2L	RR-1 ?	345	20	diad 82	23.8		1	8	2.3	13	3.8	6.1	20	5.8	33	9.6	15.4	22	6.4	24	7.0	
CPR72	2L	RR-2	120	18	27	22.5			18	15.0	7	5.8	20.8	20	16.7	6	5.0	21.7	3	2.5	6	5.0	
CPR60	2L	RR-2	123	17	53	43.1			15	12.2	6	4.9	17.1	17	13.8	7	5.7	19.5	5	4.1	5	4.1	
CPR59	2L	RR-2	194	18	19	9.8			15	7.7	7	3.6	11.3	10	5.2	49	25.3	30.4	15	7.7	3	1.5	10
CPR58	2L	RR-2	147	22	14	9.5			15	10.2	8	5.4	15.6	11	7.5	16	10.9	18.4	11	7.5	3	2.0	1
CPR57	2L	RR-2	114	27	31	27.2		2	7	6.1	4	3.5	9.6	9	7.9	8	7.0	14.9	8	7.0	4	3.5	
CPR56	2L	RR-2	150	18	57	38.0			6	4.0	24	16.0	20.0	12	8.0	4	2.7	10.7	5	3.3	4	2.7	
CPR69	2L	RR-2	230	17	140	60.9		1	4	1.7	25	10.9	12.6	18	7.8	16	7.0	14.8	7	3.0	24	10.4	
CPR101	2L	RR-2	133	19	39	29.3			15	11.3	8	6.0	17.3	10	7.5	9	6.8	14.3	7	5.3	7	5.3	
CPR55	2L	RR-2	136	16	41	30.1		1	6	4.4	17	12.5	16.9	10	7.4	10	7.4	14.7	5	3.7	10	7.4	
CPR68	2L	RR-2	115	23	18	15.7			8	7.0	5	4.3	11.3	13	11.3	3	2.6	13.9	9	7.8	6	5.2	
CPR67	2L	RR-2	226	18	122	54.0		1	26	11.5	16	7.1	18.6	21	9.3	19	8.4	17.7	12	5.3	10	4.4	
CPR136	2L	RR-2	126	17	17	13.5			23	18.3	10	7.9	26.2	11	8.7	8	6.3	15.1	10	7.9	3	2.4	
CPR54	2L	RR-2	126	17	17	13.5			23	18.3	10	7.9	26.2	11	8.7	9	7.1	15.9	9	7.1	3	2.4	
CPR53	2L	RR-2	126	17	17	13.5			23	18.3	10	7.9	26.2	11	8.7	8	6.3	15.1	9	7.1	3	2.4	
CPR52	2L	RR-2	112	17	18	16.1			17	15.2	10	8.9	24.1	15	13.4	4	3.6	17.0	3	2.7	4	3.6	
CPR51	2L	RR-2	126	17	17	13.5			23	18.3	10	7.9	26.2	11	8.7	9	7.1	15.9	10	7.9	3	2.4	
CPR50	2L	RR-2	122	17	17	13.9			22	18.0	10	8.2	26.2	11	9.0	7	5.7	14.8	9	7.4	3	2.5	
CPR49	2L	RR-2	126	17	17	13.5			23	18.3	10	7.9	26.2	11	8.7	9	7.1	15.9	9	7.1	3	2.4	
CPR48	2L	RR-2	112	17	18	16.1			17	15.2	9	8.0	23.2	15	13.4	4	3.6	17.0	3	2.7	4	3.6	

Name	Chrom.	Class	Mature AA	Signal	Start of triad	% to start	#C	#M	# H	%H	# K	%K	%H+%K	# G	%G	# A	%A	%G+%A	#P	%P	#Q	%Q	#AAP(AVL)
CPR47	2L	RR-2	101	17	28	27.7			13	12.9	10	9.9	22.8	13	12.9	5	5.0	17.8	4	4.0	4	4.0	
CPR46	2L	RR-2	112	17	13	11.6			20	17.9	10	8.9	26.8	14	12.5	7	6.3	18.8	3	2.7	4	3.6	
CPR45	2L	RR-2	112	17	13	11.6			20	17.9	7	6.3	24.1	14	12.5	7	6.3	18.8	3	2.7	4	3.6	
CPR44	2L	RR-2	112	17	13	11.6			20	17.9	7	6.3	24.1	14	12.5	7	6.3	18.8	3	2.7	4	3.6	
CPR43	2L	RR-2	112	17	13	11.6			20	17.9	7	6.3	24.1	14	12.5	7	6.3	18.8	3	2.7	4	3.6	
CPR42	2L	RR-2	106	17	13	12.3			18	17.0	7	6.6	23.6	13	12.3	7	6.6	18.9	3	2.8	3	2.8	
CPR41	2L	RR-2	112	17	13	11.6			20	17.9	7	6.3	24.1	14	12.5	8	7.1	19.6	3	2.7	3	2.7	
CPR40	2L	RR-2	112	17	13	11.6			20	17.9	7	6.3	24.1	14	12.5	7	6.3	18.8	3	2.7	4	3.6	
CPR39	2L	RR-2	111	17	13	11.7			20	18.0	6	5.4	23.4	14	12.6	8	7.2	19.8	3	2.7	4	3.6	
CPR38	2L	RR-2	107	18	12	11.2	1		18	16.8	7	6.5	23.4	11	10.3	5	4.7	15.0	3	2.8	5	4.7	
CPR37	2L	RR-2	112	17	13	11.6			19	17.0	7	6.3	23.2	14	12.5	6	5.4	17.9	3	2.7	4	3.6	
CPR66	2L	RR-2	106	16	14	13.2			19	17.9	8	7.5	25.5	12	11.3	8	7.5	18.9	3	2.8	3	2.8	
CPR145	2L	RR-2	103	17	10	9.7			13	12.6	8	7.8	20.4	14	13.6	4	3.9	17.5	3	2.9	3	2.9	
CPR36	2L	RR-2	112	17	13	11.6			22	19.6	6	5.4	25.0	15	13.4	7	6.3	19.6	3	2.7	3	2.7	
CPR35	2L	RR-2	105	18	13	12.4			17	16.2	8	7.6	23.8	11	10.5	8	7.6	18.1	3	2.9	4	3.8	
CPR65	2L	RR-2	103	17	10	9.7			13	12.6	8	7.8	20.4	14	13.6	5	4.9	18.4	2	1.9	3	2.9	
CPR34	2L	RR-2	119	18	26	21.8			18	15.1	7	5.9	21.0	21	17.6	5	4.2	21.8	3	2.5	5	4.2	
CPR64	2L	RR-2	177	16	58	32.8			32	18.1	12	6.8	24.9	12	6.8	17	9.6	16.4	10	5.6	7	4.0	1
CPR63	2L	RR-2	163	19	69	42.3			12	7.4	10	6.1	13.5	25	15.3	9	5.5	20.9	4	2.5	8	4.9	
CPR141	2L	RR-2	370	22	17	4.6		2	30	8.1	36	9.7	17.8	14	3.8	23	6.2	10.0	30	8.1	14	3.8	
CPR140	2L	RR-2	837	19	760	90.8	1		7	0.8	42	5.0	5.9	35	4.2	196	23.4	27.6	76	9.1	7	0.8	9
CPR111	2L	RR-3 ?	309	16	50	16.2		1	6	1.9	11	3.6	5.5	8	2.6	101	32.7	35.3	28	9.1	12	3.9	7
CPR61	2L	RR-1	132	18	34	25.8			2	1.5	2	1.5	3.0	11	8.3	16	12.1	20.5	14	10.6	8	6.1	
CPR62	2L	RR-1	132	18	79	59.8			3	2.3	2	1.5	3.8	12	9.1	19	14.4	23.5	12	9.1	10	7.6	
CPR110	3R	RR-2	171	17	23	13.5			14	8.2	4	2.3	10.5	6	3.5	38	22.2	25.7	14	8.2	6	3.5	2
CPR73	3R	RR-1	151	15	monad 34	22.5			8	5.3	7	4.6	9.9	11	7.3	9	6.0	13.2	11	7.3	14	9.3	
CPR74	3R	RR-1	105	17	24	22.9			1	1.0	4	3.8	4.8	8	7.6	10	9.5	17.1	7	6.7	12	11.4	
CPR151	3R	RR-1	136	17	44	32.4			6	4.4	7	5.1	9.6	13	9.6	9	6.6	16.2	12	8.8	23	16.9	
CPR75	3R	RR-1	117	17	24	20.5			1	0.9	2	1.7	2.6	8	6.8	14	12.0	18.8	13	11.1	10	8.5	
CPR133	3R	RR-1	314	17	229	72.9			1	0.3	11	3.5	3.8	94	29.9	47	15.0	44.9	13	4.1	7	2.2	
CPR153	3R	RR-1	317	17	232	73.2			1	0.3	11	3.5	3.8	96	30.3	48	15.1	45.4	13	4.1	7	2.2	
CPR76	3R	RR-1	249	19	168	67.5			4	1.6	12	4.8	6.4	29	11.6	27	10.8	22.5	26	10.4	11	4.4	
CPR77	3R	RR-1	108	18	19	17.6			1	0.9	3	2.8	3.7	9	8.3	10	9.3	17.6	8	7.4	10	9.3	
CPR78	3R	RR-1	117	20	25	21.4			1	0.9	1	0.9	1.7	10	8.5	11	9.4	17.9	16	13.7	11	9.4	
CPR79	3R	RR-1	371	15	169	45.6		1	1	0.3	6	1.6	1.9	112	30.2	49	13.2	43.4	57	15.4	15	4.0	
CPR80	3R	RR-1	228	22	111	48.7			16	7.0	5	2.2	9.2	14	6.1	21	9.2	15.4	19	8.3	42	18.4	
CPR81	3R	RR-1	114	17	37	32.5		1	1	0.9	1	0.9	1.8	14	12.3	8	7.0	19.3	14	12.3	7	6.1	
CPR82	3R	RR-2	230	17	64	27.8			15	6.5	16	7.0	13.5	7	3.0	31	13.5	16.5	23	10.0	26	11.3	
CPR107	3R	RR-2	163	17	53	32.5			17	10.4	11	6.7	17.2	6	3.7	15	9.2	12.9	14	8.6	14	8.6	
CPR83	3R	RR-2	142	17	18	12.7			10	7.0	10	7.0	14.1	6	4.2	17	12.0	16.2	10	7.0	4	2.8	
CPR108	3R	RR-2	118	17	19	16.1			7	5.9	7	5.9	11.9	5	4.2	19	16.1	20.3	8	6.8	3	2.5	
CPR84	3R	RR-2	118	17	19	16.1			7	5.9	7	5.9	11.9	5	4.2	19	16.1	20.3	0.8	0.7	3	2.5	

Name	Chrom.	Class	Mature AA	Signal	Start of triad	% to start	#C	#M	# H	%H	# K	%K	%H+%K	# G	%G	# A	%A	%G+%A	#P	%P	#Q	%Q	#AAP(AVL)
CPR85	3R	RR-2	160	20	17	10.6			12	7.5	12	7.5	15.0	9	5.6	6	3.8	9.4	9	5.6	7	4.4	
CPR155	3R	RR-2	123	28	17	13.8			1	0.8	9	7.3	8.1	10	8.1	5	4.1	12.2	3	2.4	2	1.6	
CPR156	3R	RR-2	108	17	29	26.9			21	19.4	7	6.5	25.9	9	8.3	6	5.6	13.9	3	2.8	3	2.8	
CPR148	3R	RR-2	115	17	27	23.5			21	18.3	6	5.2	23.5	9	7.8	6	5.2	13.0	7	6.1	14	12.2	
CPR86	3R	RR-2	115	17	27	23.5			22	19.1	5	4.3	23.5	9	7.8	4	3.5	11.3	7	6.1	14	12.2	
CPR87	3R	RR-2	115	17	27	23.5			21	18.3	5	4.3	22.6	10	8.7	5	4.3	13.0	6	5.2	13	11.3	
CPR88	3R	RR-2	108	16	27	25.0			19	17.6	5	4.6	22.2	9	8.3	5	4.6	13.0	6	5.6	10	9.3	
CPR89	3R	RR-2	115	17	27	23.5			22	19.1	5	4.3	23.5	9	7.8	6	5.2	13.0	7	6.1	13	11.3	
CPR90	3R	RR-2	115	17	27	23.5			21	18.3	5	4.3	22.6	9	7.8	6	5.2	13.0	7	6.1	13	11.3	
CPR91	3R	RR-2	107	17	27	25.2			19	17.8	5	4.7	22.4	9	8.4	4	3.7	12.1	5	4.7	13	12.1	
CPR150	3R	RR-2	176	17	94	53.4			8	4.5	9	5.1	9.7	7	4.0	22	12.5	16.5	16	9.1	21	11.9	
CPR92	3R	RR-2	208	17	75	36.1			38	18.3	5	2.4	20.7	9	4.3	33	15.9	20.2	15	7.2	10	4.8	2
CPR93	3R	RR-2	214	17	67	31.3			39	18.2	5	2.3	20.6	8	3.7	36	16.8	20.6	16	7.5	9	4.2	2
CPR94	3R	RR-2	214	17	67	31.3			39	18.2	5	2.3	20.6	9	4.2	36	16.8	21.0	16	7.5	9	4.2	2
CPR109	3R	RR-2	214	17	67	31.3			38	17.8	5	2.3	20.1	8	3.7	35	16.4	20.1	17	7.9	10	4.7	2
CPR95	3R	RR-2	218	17	75	34.4			40	18.3	5	2.3	20.6	9	4.1	37	17.0	21.1	17	7.8	10	4.6	2
CPR96	3R	RR-2	210	15	67	31.9			38	18.1	5	2.4	20.5	9	4.3	34	16.2	20.5	16	7.6	10	4.8	2
CPR97	3R	RR-2	222	17	75	33.8			39	17.6	5	2.3	19.8	9	4.1	37	16.7	20.7	17	7.7	11	5.0	1
CPR149	3R	RR-2	107	17	27	25.2			20	18.7	5	4.7	23.4	9	8.4	4	3.7	12.1	5	4.7	13	12.1	
CPR132	3R	RR-2	342	15	73	21.3			12	3.5	2	0.6	4.1	9	2.6	14	4.1	6.7	11	3.2	55	16.1	
CPR131	3R	RR-2	170	17	57	33.5			12	7.1	10	5.9	12.9	8	4.7	19	11.2	15.9	13	7.6	15	8.8	
CPR98	3R	RR-2	176	17	94	53.4			8	4.5	10	5.7	10.2	7	4.0	21	11.9	15.9	16	9.1	22	12.5	
CPR142	3R	RR-2	218	17	71	32.6			40	18.3	5	2.3	20.6	9	4.1	38	17.4	21.6	16	7.3	9	4.1	1
CPR99	3R	RR-2	214	17	67	31.3			39	18.2	5	2.3	20.6	9	4.2	37	17.3	21.5	16	7.5	9	4.2	2
CPR100	3R	RR-2	214	17	67	31.3			38	17.8	5	2.3	20.1	9	4.2	35	16.4	20.6	16	7.5	10	4.7	1
CPR112	3L	RR-3 ?	147	17	6	4.1			4	2.7	9	6.1	8.8	6	4.1	12	8.2	12.2	11	7.5	6	4.1	
CPR143	3L	RR-2	470	22	132	28.1		3	12	2.6	19	4.0	6.6	27	5.7	66	14.0	19.8	42	8.9	48	10.2	1
CPR113	3L	RR-1	305	17	118	38.7			6	2.0	3	1.0	3.0	35	11.5	29	9.5	21.0	41	13.4	56	18.4	
CPR147	UNKN	RR-2	163	18	80	49.1		1	1	0.6	2	1.2	1.8	23	14.1	7	4.3	18.4	12	7.4	9	5.5	
CPR146	UNKN	RR-2	134	19	54	40.3		1	4	3.0	7	5.2	8.2	15	11.2	8	6.0	17.2	16	11.9	12	9.0	
CPR152	UNKN	RR-2	289	28	83	28.7		1	58	20.1	7	2.4	22.5	35	12.1	8	2.8	14.9	16	5.5	6	2.1	
			Mature AA	Signal		% to start	#C	#M		%H		%K	%H+%K		%G		%A	%G+%A		%P		%Q	#AAP(AVL)
MEAN			169	17.8		0.3	0.03	0.38		7.8		5.0	12.8		9.2		10.4	19.6		6.9		5.7	0.9

Tandem arrays are shown in alternating shades of gray. Sequence clusters are highlighted in color. RR Class was determined at cuticleDB; those with a (?) were manually assigned. Six proteins in triad column had only one (monad) or two (diad) aromatic residues at the start of the R&R Consensus. Triad start was measured in the mature protein.
