# Supplementary material for: Annotation and analysis of a large cuticular protein family with the R&R Consensus in Anopheles gambiae
Source: BMC Genomics. 2008 Jan 18;9:22. doi: 10.1186/1471-2164-9-22 (PMC2259329; doi:10.1186/1471-2164-9-22)
Supplement: Additional file 5 — Supplementary Table 5. Comparison of An. gambiae and D. melanogaster orthologs. [file 1471-2164-9-22-S5.PDF]

Supplementary Table 5. Comparison of *An. gambiae* and *D. melanogaster* orthologs.

	% similarity																						
Ortholog pairs[a]	total	cons.[b]	Chrom.	Mature AA	Signal	Triad start	% to start	#C	#M	# H	%H	# K	%K	# G	%G	# A	%A	%G+A	#P	%P	#AAP	#Q	%Q
**AgamCPR130**	53	70	X	334	20	20	6.0			38	11.4	14	4.2	26	7.8	76	22.8	30.5	29	8.7	1	12	3.6
**DmelCpr92F**			3R	364	17	20	5.5			54	14.8	8	2.2	41	11.3	68	18.7	29.9	25	6.9	1	15	4.1
**AgamCPR129**	29	70	X	223	20	diad 55	24.7		3	2	0.9	15	6.7	13	5.8	11	4.9	10.8	22	9.9		9	4
**DmelCpr11A**			X	254	16	diad 51	20.1		1	3	1.2	15	5.9	41	16.1	11	4.3	20.5	15	5.9		14	5.5
**AgamCPR127**	55	80	X	208	16	23	11.1			6	2.9	8	3.8	15	7.2	15	7.2	14.4	26	12.5		23	11
**DmelCpr97Eb**			3R	215	20	24	11.2			5	2.3	12	5.6	16	7.4	11	5.1	12.6	30	14.0		20	9.3
**AgamCPR126**	47	98	X	338	19	40	11.8			12	3.6	17	5.0	23	6.8	36	10.7	17.5	54	16.0	4	43	13
**DmelCpr97Ea**			3R	344	19	50	14.5			5	1.5	10	2.9	30	8.7	32	9.3	18.0	56	16.3	1	41	12
**AgamCPR125**	66	84	X	211	16	monad 24	11.4			6	2.8	6	2.8	17	8.1	23	10.9	19.0	33	15.6	2	25	12
**DmelCpr100A**			3R	225	16	monad 25	11.1			5	2.2	6	2.7	16	7.1	18	8.0	15.1	34	15.1		42	19
**AgamCPR9**	50	75	2R	182	17	33	18.1			10	5.5	10	5.5	9	4.9	23	12.6	17.6	13	7.1		9	4.9
**DmelCpr57A**			2R	169	15	17	10.1			15	8.9	5	3.0	10	5.9	30	17.8	23.7	10	5.9		15	8.9
AgamCPR114	48	84	2R	147	17	59	40.1			13	8.8	8	5.4	10	6.8	25	17.0	23.8	14	9.5	2	1	0.7
DmelCpr64Ac			3L	170	18	74	43.5			4	2.4	11	6.5	13	7.6	35	20.6	28.2	17	10.0	4	3	1.8
AgamCPR116	55	84	2R	107	21	21	19.6					2	1.9	8	7.5	15	14.0	21.5	12	11.2		5	4.7
DmelCpr64Ab			3L	101	19	23	22.8			1	1.0	2	2.0	8	7.9	14	13.9	21.8	11	10.9		5	5
AgamCPR124	51	88	2R	226	18	138	61.1					6	2.7	5	2.2	73	32.3	34.5	26	11.5	10	7	3.1
DmelCpr64Ad			3L	228	19	127	55.7			1	0.4	6	2.6	12	5.3	64	28.1	33.3	35	15.4	12	4	1.8
**AgamCPR138**	31	51	2L	385	18	303	78.7	2	5	10	2.6	32	8.3	40	10.4	20	5.2	15.6	47	12.2		15	3.9
**DmelI(3)mbn**			3L	629	24	551	87.6	2	4	12	1.9	38	6.0	89	14.1	59	9.4	23.5	45	7.2	1	25	4

	% similarity																						
Ortholog pairs[a]	total	cons.[b]	Chrom.	Mature AA	Signal	Triad start	% to start	#C	#M	# H	%H	# K	%K	# G	%G	# A	%A	%G+A	#P	%P	#AAP	#Q	%Q
AgamCPR135	50	90	2L	239	17	125	52.3			3	1.3	9	3.8	12	5.0	20	8.4	13.4	25	10.5		59	25
DmelCpr66D			3L	251	19	139	55.4			5	2.0	12	4.8	14	5.6	15	6.0	11.6	20	8.0		57	23
AgamCPR70	73	98	2L	123	16	38	30.9		9	11	8.9	7	5.7	7	5.7	17	13.8	19.5	12	9.8	1	3	2.4
DmelCpr66Ca			3L	122	16	36	29.5			14	11.5	6	4.9	7	5.7	17	13.9	19.7	11	9.0	1	2	1.6
AgamCPR144	39	60	2L	569	19					13	2.3	23	4.0	76	13.4	37	6.5	19.9	59	10.4		23	4
DmelCpr73D			3L	564	27				3	14	2.5	12	2.1	101	17.9	43	7.6	25.5	36	6.4		13	2.3
AgamCPR60	70	95	2L	123	17	53	43.1			15	12.2	6	4.9	17	13.8	7	5.7	19.5	5	4.1		5	4.1
DmelCpr66Cb			3L	145	17	72	49.7			26	17.9	6	4.1	11	7.6	7	4.8	12.4	8	5.5		4	2.8
AgamCPR59	61	82	2L	194	18	19	9.8			15	7.7	7	3.6	10	5.2	49	25.3	30.4	15	7.7	10	3	1.5
DmelCpr62Bc			3L	162	18	36	22.2			22	13.6	4	2.5	16	9.9	34	21.0	30.9	11	6.8	5	2	1.2
AgamCPR58	56	90	2L	147	22	14	9.5			15	10.2	8	5.4	11	7.5	16	10.9	18.4	11	7.5	1	3	2
DmelCpr62Bb			3L	173	21	14	8.1			14	8.1	7	4.0	13	7.5	22	12.7	20.2	15	8.7		9	5.2
AgamCPR141	32	76	2L	370	22	17	4.6		2	30	8.1	36	9.7	14	3.8	23	6.2	10.0	30	8.1		14	3.8
DmelCpr76Bc			3L	404	20	36	8.9		6	28	6.9	37	9.2	24	5.9	20	5.0	10.9	36	8.9		19	4.7
AgamCPR140	34	82	2L	837	19	760	90.8	1		7	0.8	42	5.0	35	4.2	196	23.4	27.6	76	9.1	9	7	0.8
DmelCpr76Bd			3L	1211	18	1136	93.8			38	3.1	65	5.4	115	9.5	215	17.8	27.3	98	8.1	17	27	2.2
AgamCPR132	25	50	3R	342	15	73	21.3			12	3.5	2	0.6	9	2.6	14	4.1	6.7	11	3.2		55	16
DmelCry			2L	457	20	57	12.5		1	1	0.2	6	1.3	11	2.4	33	7.2	9.6	12	2.6		67	15
AgamCPR147			UNKN	163	18	80	49.1		1	1	0.6	2	1.2	23	14.1	7	4.3	18.4	12	7.4		9	5.5
DmelCpr56F	49	79	2R	198	19	109	55.1		1	1	0.5	5	2.5	40	20.2	12	6.1	26.3	20	10.1		17	8.6

	% similarity																						
Ortholog pairs[a]	total	cons.[b]	Chrom.	Mature AA	Signal	Triad start	% to start	#C	#M	# H	%H	# K	%K	# G	%G	# A	%A	%G+A	#P	%P	#AAP	#Q	%Q
AgamCPR146	50	76	UNKN	134	19	54	40.3		1	4	3.0	7	5.2	15	11.2	8	6.0	17.2	16	11.9		12	9
DmelCpr50Cb			2R	155	23	67	43.2		1	4	2.6	6	3.9	26	16.8	15	9.7	26.5	25	16.1		7	4.5
AgamCPR152			UNKN	289	28	83	28.7		1	58	20.1	7	2.4	35	12.1	8	2.8	14.9	1.6	0.6		6	2.1
Dmelresilin	31	76	2R	603	17	328	54.4		1	1	0.2	5	0.8	210	34.8	29	4.8	39.6	64	10.6		32	5.3

[a]Names of RR-1 proteins are bolded in green; [b]Consensus; Pairs of cells that are highlighted in gray have percentages that differ by more than 50%.
