# Supplementary material for: Annotation and analysis of a large cuticular protein family with the R&R Consensus in Anopheles gambiae
Source: BMC Genomics. 2008 Jan 18;9:22. doi: 10.1186/1471-2164-9-22 (PMC2259329; doi:10.1186/1471-2164-9-22)
Supplement: Additional file 6 — Supplementary Table 6. Position-specific scoring matrix of the motif identified at the C-terminus of most RR-2 genes on chromosome 2L. [file 1471-2164-9-22-S6.PDF]

Supplementary Table 6. Position-specific scoring matrix of the motif identified at the C-terminus of most RR-2 genes on chromosome 2L.

ALPHABET=ACDEFGHIKLMNPQRSTVWY

log-odds matrix: alength= 20 w= 16

149	-39	-330	-114	-512	47	135	-683	-91	-584	-432	-264	-733	-229	-248	-478	-488	-734	-468	44
-659	57	25	-207	-342	174	214	-641	-468	-504	-342	-179	-145	-136	225	-227	-439	-686	-328	-46
-710	3	-384	-218	-239	28	271	-687	-584	-556	-418	-291	45	-534	-418	-512	-531	-741	-276	-132
-360	18	-103	-499	-532	234	-12	-763	-532	-680	-534	-232	218	-600	-472	-144	-477	-767	-535	45
-579	214	-303	-225	-147	-102	269	-477	-470	-322	302	-122	-566	-241	-148	38	-354	-301	-134	-106
-47	73	-465	-595	-496	369	-826	-659	-577	-620	-422	-414	-724	-691	-436	-483	-532	-679	-407	-812
-125	-73	-311	333	38	-605	-241	-652	-106	-553	-412	-349	-728	-508	-399	183	-522	-700	-305	59
-1009	-240	-859	-1022	-768	-862	-1128	-1043	-994	-895	-840	-874	-980	-1037	-800	395	-900	-1077	-669	-1103
-927	-68	-773	-845	-107	-849	-182	-657	-786	-527	-432	-611	-914	-771	-593	-760	-727	-758	307	348
166	150	-686	-758	-543	-526	-966	-690	-769	-632	-451	-103	-741	-91	-589	148	-26	169	-533	-926
-674	-33	-292	-480	-553	-508	-693	-747	291	-653	-510	474	-729	-555	-350	-145	-174	-788	-526	-788
-573	173	-623	-630	-241	58	-804	377	-596	265	-125	-510	-732	-246	-457	-157	-411	45	-312	-651
-274	-16	416	-59	-477	-245	-142	-651	130	-562	-400	293	-678	-85	40	-420	-436	-700	-462	-142
-706	-56	-602	-515	-544	-669	-240	-616	226	199	-353	-429	-769	332	-130	-580	-515	-682	-420	-763
-918	-62	-755	-227	198	-842	267	-652	-778	-74	-425	-590	-906	-744	-576	-748	-716	-751	-158	129
-867	-46	-687	-770	199	-803	95	-636	-688	-167	-411	237	-880	96	33	-256	-686	-733	-158	247
