# Supplementary material for: Annotation and analysis of a large cuticular protein family with the R&R Consensus in Anopheles gambiae
Source: BMC Genomics. 2008 Jan 18;9:22. doi: 10.1186/1471-2164-9-22 (PMC2259329; doi:10.1186/1471-2164-9-22)
Supplement: Additional file 7 — Supplementary Table 7. Oligonucleotide primers used for obtaining RACE products and RT-PCR products from selected genes. [file 1471-2164-9-22-S7.PDF]

Supplementary Table 7. Oligonucleotide primers used for obtaining RACE products and cDNAs from selected genes.

Targeted Gene	Region Covered	Technique	Primer[a]	
CPR7	full coding	RT-PCR	AGCPR7UB	AGCPR7DB
CPR10	full coding	RT-PCR	AGCPR10UC	AGCPR10DA
CPR11	partial coding	RT-PCR	AGCPR11UA	AGCPR11DA
CPR12	full coding	RT-PCR	AGCPR12UA	AGCPR12DA
CPR68	5′ + 89% coding	5' RACE	GR5	AGCPR68DC
			GR5N	AGCPR68DD
CPR79	full coding	RT-PCR	AGCPR79UA	AGCPR79DA
*CPR115**	5′UTR + 93% coding	5' RACE	GR5	AGCPR154DA
			GR5N	AGCPR154DB
*CPR120**	5′UTR + 93% coding	5' RACE	GR5	AGCPR154DA
			GR5N	AGCPR154DB
CPR127	5′UTR to 3rd exon	5' RACE	GR5	AGCPR127DA
			GR5N	AGCPR127DB
CPR129	full coding	RT-PCR	AGCPR129UA	AGCPR129DA
CPR130	5′UTR to 2nd exon	5' RACE	GR5	AGCPR130DC
			GR5N	AGCPR130DD
CPR134	5′UTR + full coding	5' RACE	GR5	AGCPR134DB
			GR5N	AGCPR134DA
CPR137	90% 5'UTR	5' RACE	GR5	AGCPR137DB
			GR5N	AGCPR137DA
	full coding + 3′UTR	3' RACE	AGCPR137UA	GR3
			AGCPR137UB	GR3N
CPR138	5′ UTR+ full coding	5' RACE	GR5	AGCPR138DB
			GR5N	AGCPR138DA
CPR139	5′UTR + 75% coding	5' RACE	GR5	AGCPR139DA
			GR5N	AGCPR139DB
CPR140	14% coding + 3′UTR	3' RACE	AGCPR140UB	RPAP
CPR143	full coding	RT-PCR	AGCPR143UA	AGCPR143DA
CPR144	partial cDNA	RT-PCR	AGCPR144UD	AGCPR144DD
	5'UTR to 3rd exon	5' RACE	GR5	AGCPR144DB
			GR5N	AGCPR144DD
CPR152	5'UTR to 3rd exon	5' RACE	GR5	AGCPR152DA
			GR5N	AGCPR152DB
	3rd exon to 3'UTR	3' RACE	AGCPR152UB	RPAP
*CPR154**	5′UTR + 93% coding	5' RACE	GR5	AGCPR154DA
			GR5N	AGCPR154DB
CPR155	5'UTR + 81% of coding	5' RACE	GR5	AGCPR155DA
			GR5N	AGCPR155DB
gene # test	shared on AAAB01008851		2RgrpUA	2RgrpDA

Primer	Sequence 5'-3′
AGCPR7UB	TTCGCGAGTTTAAAACACACAGTTTTC
AGCPR7DB	CCTCCAACATCAGCGCATCC
AGCPR10UC	TCCAGCGTCAGCAACACTTA
AGCPR10DA	TTTTGTGTACGGTGGCATTTTATTGA
AGCPR11UA	CCGTGAAAAGTAAACACAGCA
AGCPR11DA	AACCTACTGGCGAATGATGC
AGCPR12UA	CCGTCGTCGTCAATTCATTA
AGCPR12DA	CAAGTTTCCAATCCCAGCAG
AGCPR12UA	CCGTCGTCGTCAATTCATTA
AGCPR68DC	TATGGACGCCCTGCACGAA
AGCPR68DD	CCAAAGTGGCTGCGGAGGA
AGCPR79UA	GTGTCCGTGCTGCGTTGGTG
AGCPR79DA	CGTTTCGCTTCAATTTCGTTGG
AGCPR127DA	TGGACGGTCTGTTTGGATTTGG
AGCPR127DB	GCGGCGGATAGTTGCTGTTG
AGCPR129UA	TGTTGCACGGTTGTGGTGTG
AGCPR129DA	GCCGGTGCCCCTTTAATCCT
AGCPR130DC	CGTGCCCGACACCTGGAA
AGCPR130DD	CCGCCGTGTACTTCACGCTCT
AGCPR134DA	GTTGTTGATGCATTCCTTCCCAAA
AGCPR134DB	TGTCTTCCTGTTTGCTGTCGTTGA
AGCPR137UA	CGCAACCCGCTCAATCACACCTTT
AGCPR137UB	TCATCATGAACCTTATCGGCGTGGT
AGCPR137DA	CCATTTTGGACATCGTTTTGCCTACC
AGCPR137DB	TTCCTGCCCGTGAACACTTTCGTAG
AGCPR138DA	TTACAAATGGGAGGGAGGCAGTGTT
AGCPR138DB	CTCGCTTTTGAATGCTCCATCCTCA
AGCPR139DA	TGGTTTTCGATTTGGCTTCTTACTGC
AGCPR139DB	TCTCCAGGAAATCGTCGAACCAA
AGCPR140UB	GTTATGCTGCCGCTGCTCCT
AGCPR143UA	GGGTTCACCCTGTGGATGTGC
AGCPR143DA	AGATAGAAAGATCATTACAACCACCAAACG
AGCPR144UD	GCCAGCGGTAACCCGAAAAC
AGCPR144DB	CCGAACCACTCGGTCCACTC
AGCPR144DD	TCCACTCTCGAACGCGACCT
AGCPR152UB	CCAGATGGTCGTCGTCAGATTGTAG
AGCPR152DA	GCGCATGTTCATGGGGATGTT
AGCPR152DB	TTCATGGTGGTGGGGATGCT
AGCPR154DA	AGGAGCGTAGGGGGCAGCAA
AGCPR154DB	AGCGGAGCAGCGATCTTGG
AGCPR155DA	TGTGAATCGCAACCCAAAGACAC
AGCPR155DB	CAATCACGGGTTCTGTTCTGATTTG
2RgrpUA	GTGAATAAGATTTACTGAGAC
2RgrpDA	GGCGAACTGTAAAAGAATC

[a]GR5, GR5N, GR3, GR3N represent Invitrogen GeneRacer® 5', 5' Nested, 3', 3' Nested primers, respectively. RPAP is the Roche PCR anchor primer.
*Primers for *CPR154* also produced RACE products that confirmed *CPR115* and *CPR120*.
5′RACE revealed that *CPR68* and *CPR127* had untranslated first exons.
